# Microalgae-derived Co_3_O_4_ nanomaterials for catalytic CO oxidation†

**DOI:** 10.1039/d4ra00343h

**Published:** 2024-02-05

**Authors:** Agnieszka Sidorowicz, Nevzat Yigit, Thomas Wicht, Michael Stöger-Pollach, Alessandro Concas, Roberto Orrù, Giacomo Cao, Günther Rupprechter

**Affiliations:** a Interdepartmental Centre of Environmental Engineering and Sciences, University of Cagliari 09123 Cagliari Italy giacomo.cao@unica.it; b Institute of Materials Chemistry, TU Wien Getreidemarkt 9/BC 1060 Vienna Austria guenther.rupprechter@tuwien.ac.at; c University Service Center for Transmission Electron Microscopy, TU Wien Wiedner Hauptstr. 8-10 1040 Vienna Austria

## Abstract

Efficient carbon monoxide oxidation is important to reduce its impacts on both human health and the environment. Following a sustainable synthesis route toward new catalysts, nanosized Co_3_O_4_ was synthesized based on extracts of microalgae: *Spirulina platensis*, *Chlorella vulgaris*, and *Haematococcus pluvialis*. Using the metabolites in the extract and applying different calcination temperatures (450, 650, 800 °C) led to Co_3_O_4_ catalysts with distinctly different properties. The obtained Co_3_O_4_ nanomaterials exhibited octahedral, nanosheet, and spherical morphologies with structural defects and surface segregation of phosphorous and potassium, originating from the extracts. The presence of P and K in the oxide nanostructures significantly improved their catalytic CO oxidation activity. When normalized by the specific surface area, the microalgae-derived catalysts exceeded a commercial benchmark catalyst. *In situ* studies revealed differences in oxygen mobility and carbonate formation during the reaction. The obtained insights may facilitate the development of new synthesis strategies for manufacturing highly active Co_3_O_4_ nanocatalysts.

## Abstract 2

Sustainable nanosized Co_3_O_4_ catalysts, derived from microalgae extracts and post-synthesis treatments, were employed for oxidative CO removal.

## Graphical abstract

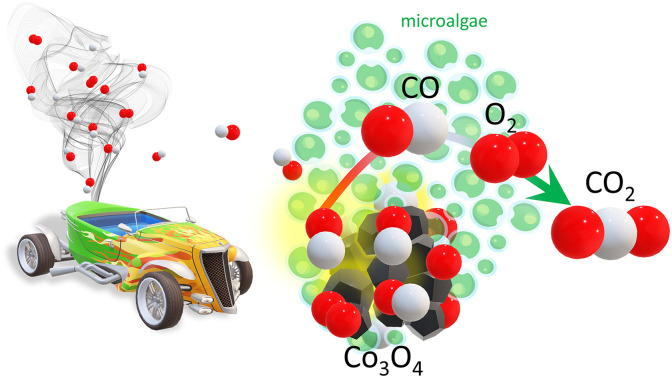

## 1 Introduction

Carbon monoxide is a by-product of incomplete fossil fuel combustion that can give rise to serious health issues such as hypoxia, in addition to environmental problems such as photochemical smog.^1^ Rapid urban development and industrialization led to a sharp rise in CO emissions generated by metallurgy, thermal power plants, petrochemical industries, coal mines, and other fields.^2,3^ In the transport sector, during the cold start of an engine, when the automotive three-way-catalyst does not yet function, mainly CO is discharged into the atmosphere.^4^ Hence, a huge number of studies focused on CO elimination, with catalytic CO oxidation to less toxic CO_2_ being the most effective and economical method. Furthermore, carbon monoxide oxidation in the recirculated flue gas of an internal combustion engine also increases the combustion efficiency, thus lowering the fossil fuel consumption.^5,6^ Apart from well-established noble metal catalysts, cobalt(ii,iii) oxide nanomaterials (Co_3_O_4_ NMs) have received considerable attention due to their abundance, low production costs, and excellent low-temperature catalytic performance.^7–12^

Despite progress and improvements in Co_3_O_4_ catalyst design, the synthesis methods are usually chemical or physical, while a biological approach has not yet been studied extensively. The typical synthesis routes often involve expensive and hazardous chemicals and procedures that counterproductively may impose negative effects on the environment. As an alternative, more environmentally friendly procedures are gaining importance. Recently, pollen has been used as a bio-template in catalyst preparation, providing a porous structure to deposit Co_3_O_4_,^13^ but the effect of the extract on Co_3_O_4_ synthesis was not studied. Organisms possess a variety of metabolites that can be employed as reducing agents, also featuring economic and environmentally-friendly aspects.^14,15^ Among various organisms, microalgae are recently gaining more attention due to the quick production of nutrient-rich biomass, as their CO_2_ fixation rate during photosynthesis is higher than that of terrestrial plants.^16^ Al Jahdaly *et al.* demonstrated the potential of marine red algae extract (*Grateloupia sparsa*) for Co_3_O_4_ NMs preparation, which were then used as electrode for energy storage applications.^17^ Their remarkable performance and stability indicated the potential of algal extract to fabricate high-value Co_3_O_4_ NMs *via* a sustainable strategy. However, the Co_3_O_4_ NMs synthesized using biological extract have not yet been tested for CO oxidation.

As mentioned, current research typically focused on Co_3_O_4_ NMs synthesized *via* chemical or physical approaches, combined with characterization and catalytic activity testing.^7–12,18–20^ Several spectroscopic and theoretical studies aimed at identifying the CO oxidation reaction mechanism on Co_3_O_4_ NMs.^8,21–27^ While Pollard *et al.* suggested CO adsorption on Co^2+^, other (theoretical) studies suggested CO adsorption on Co^3+^ ions as active sites.^21^ The abundance of surface Co^3+^ ions was also made responsible for cryogenic (−77 °C) CO oxidation on Co_3_O_4_ nanorods, mainly exposing (110) planes.^12^ The morphology-dependent activity of Co_3_O_4_ NM surfaces was determined as (110) > (100) > (111) by a theoretical study.^22^ However, Teng *et al.* reported that plate-like Co_3_O_4_ with exposed (111) planes was more active than cubic- or rod-shaped Co_3_O_4_, which exhibited (100) and (110) planes, respectively.^23^ Recently, the involvement of other crystal planes was studied *via* synthesizing various other morphologies such as straw-like nanorods, flower-like nanosheets, or honeycomb- and raspberry-shaped nanoparticles.^24,25^ The results revealed a complex surface chemistry with the reactivity depending on the exposed morphologies, crystal sizes and crystal facets.

Another strategy to study the relationship between structure and activity is introducing heteroatoms to induce structural defects. Lou *et al.* reported that doping by heteroatoms with larger ionic radii changed the lattice parameter and promoted reducibility as well as oxygen mobility.^27^ The doping effect was investigated for potassium, phosphorous, chlorine, and alkali metals (Na, K, and Li),^28–31^ revealing structural defects with either enhanced or inhibited activity. However, the combined effect of multiple elements has not been studied so far.

In the study presented herein, methanolic extracts of three different microalgae were used to synthesize Co_3_O_4_ NMs: *Spirulina platensis* (blue-green algae), *Chlorella vulgaris* (green algae), and *Haematococcus pluvialis* (green algae). These species are well-known and commonly cultivated for various purposes, including nutritional supplements, food additives, and bioactive compound production. To the best of our knowledge, this is the first work dealing with Co_3_O_4_ NMs synthesized from extracts of *C. vulgaris* and *H. pluvialis*. The involvement of metabolites was examined by ultraviolet-visible (UV-Vis) and attenuated total reflection Fourier transform infrared spectroscopy (ATR-FTIR), applied to the extract used for the synthesis, as well as after synthesis. The obtained Co_3_O_4_ NMs were characterized by X-ray diffraction (XRD), Brunauer–Emmett–Teller (BET), UV-Vis, scanning electron microscopy (SEM), transmission electron microscopy (TEM), electron energy loss spectroscopy (EELS), X-ray photoelectron spectroscopy (XPS), and universal ATR-FTIR (UATR). The availability of oxygen species was evaluated by temperature programmed desorption O_2_-TPD and reduction (H_2_-TPR). The catalysts were then tested for CO oxidation to establish a connection between structure, composition and activity. The catalytic performance was determined in a continuous-flow reactor at room temperature (RT), and the mechanism was studied by *in situ* diffuse reflectance infrared Fourier transform spectroscopy (DRIFTS) and differential scanning calorimetry (DSC). Altogether, the results illustrate the potential of algae extract components for Co_3_O_4_ NM synthesis as catalyst for CO oxidation, providing the basis for further studies.

## 2 Experimental

### 2.1 Extract preparation

*S. platensis* (courtesy of TOLO Green, Arborea, Italy) and *C. vulgaris* (CCALA 902) cultures were cultivated in Modified Zarrouk Medium and Bold Basal Medium (BBM), respectively, at 25 °C under stirring (250 RPM IKA® RH Digital Magnetic Stirrer) and 30 μmol m^−2^ s^−1^ photosynthetic photon flux density (PPFD) in 12/12 light cycle. *H. pluvialis* (CCALA 840) culture was grown in Modified Optimal Haematococcus Medium (OHM) at 31 °C with stirring (Zetalab®, Italy) and 50 μmol m^−2^ s^−1^ PPFD in 12/12 light cycle. The culture was supplied with 10 mM CH_3_COONa twice a week to improve growth and then daily for two weeks to increase the astaxanthin content. Modified Zarrouk medium was prepared according to the composition in Table S1† with pH adjustment to 9.0, thereafter autoclaved, and, after solution cool down, K_2_SO_4_ and MgSO_4_ were added to the medium as axenic. Similarly, BBM and Modified OHM media were prepared as reported in Tables S2 and S3,† the pH set to 6.2 and 8.0 for BBM and Modified OHM media, respectively, and the media were autoclaved.

The cultures were cultivated until reaching optical density at 750 nm (OD_750_) around 0.6, 0.4, and 0.3 for *S. platensis*, *C. vulgaris*, and *H. pluvialis*, respectively. Then, the biomass was separated from the media by centrifugation at 4 °C (1500 RPM for *S. platensis*, 2000 RPM for *C. vulgaris*, and 1200 RPM for *H. pluvialis*) and dried at room temperature for 7 days. The samples were imaged with an optical microscope (Fig. S2†). The obtained biomass was weighted, yielding 9 g of *S. platensis*, 2.75 g of *C. vulgaris*, and 2.5 g of *H. pluvialis* used for extract preparation.

### 2.2 Catalyst preparation

**Fig. 1:**
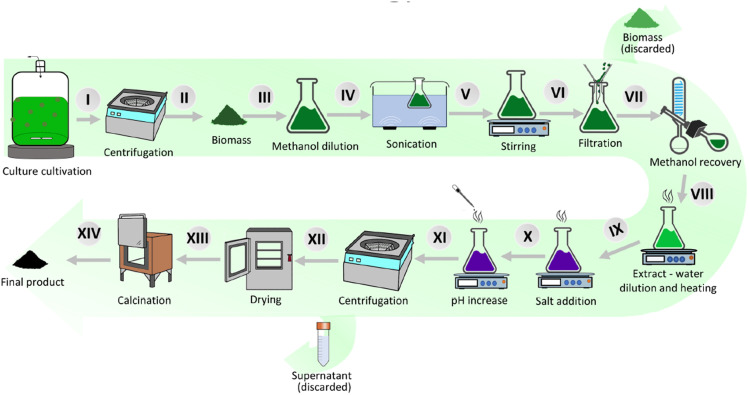
Methodology of Co_3_O_4_ nanomaterial synthesis from microalgae.

The microalgae-based Co_3_O_4_ NMs synthesis procedure is depicted in Fig. 1. First, the obtained algal biomass (Fig. 1I and II) was suspended in methanol (Merck® LiChrosolv® hypergrade) (Fig. 1III) according to the weight (540 ml for *S. platensis*, 165 ml for *C. vulgaris*, 150 ml for *H. pluvialis*). The flasks were sonicated for 30 min (Soltec® Sonica® 2400 ETH S3) (Fig. 1IV) and then stirred at 250 RPM (Fig. 1V) for 30 min. Next, the biomass was removed using standard filtration paper (Whatman®) (Fig. 1VI), and the solutions were evaporated using a rotary evaporator (Buchi Rotavapor™ R-210 Rotary Evaporator System) to remove about 70% of methanol (Fig. 1VII). Finally, the concentrated extracts were diluted using MiliQ H_2_O (Millipore®, Milan, Italy) to 1080 ml, 330 ml, and 300 ml for *S. platensis*, *C. vulgaris*, and *H. pluvialis*, respectively (Fig. 1VIII).

The prepared extracts were heated to 85 °C under stirring at 250 RPM (IKA® RH Digital Magnetic Stirrer), with an extract sample taken and labeled as “extract before synthesis” (Fig. 1IX). In the next step, 13.52 g, 4.29 g, or 3.90 g of cobalt(ii) chloride hexahydrate (Carlo Erba®, Italy) was added to *S. platensis*, *C. vulgaris*, and *H. pluvialis* extract, respectively. After 15 min, the pH was raised to 8 (*S. platensis* and *C. vulgaris*) and 10 (*H. pluvialis*) using 1.25 M NaOH. After salt addition, the flasks were continuously heated and stirred for 1.5 h. Then, the solutions were removed from the heat source and left to cool down to RT. The obtained nanomaterials were separated from the supernatant by centrifugation at 4 °C with 4000 RPM (Heraeus® Megafuge® 1.0R) and dried at 80 °C for 24 h. The supernatant was labeled as “extract after synthesis” (Fig. 1XII). Next, the products were calcined in air in a muffle furnace (Gelman Instrument®) for 3 h at 450, 650 or 800 °C. Finally, the nanomaterials were stored at RT in the dark.

The samples were labeled according to the species used for the synthesis (*Spirulina platensis* – SP, *Chlorella vulgaris* – CH, *Haematococcus pluvialis* – HA) and their calcination temperature. A commercially available Co_3_O_4_ NM (Sigma-Aldrich®, Italy) after 400 °C calcination^8^ was labeled as SA400.

### 2.3 Extract characterization

The microalgae samples were observed using a polarized light (PL) optical microscope (Leica DM 2000, Leica Microsystems, Heerburg, Switzerland) coupled to a Leica EC3 digital camera (Leica Microsystems). One drop of suspended microalgae was placed on a glass slide, and a glass coverslip was placed above it. Images were acquired using LeicaSuite LAS EZ software.

UV-Vis measurements were performed on a UV-1600PC spectrophotometer at RT. The extract samples before and after synthesis were measured in the 200–1000 nm wavelength range. The nanomaterials were suspended in deionized water (10 mg ml^−1^) and sonicated for 15 min, followed by measurement from 300 to 1000 nm. The direct bandgap energy was calculated from the Tauc relation (eqn (1)):

1 (*αhν*)^2^ = (*hν* − *E*_g_)

where *α* is the molar extinction coefficient, *h* is Plank's constant, *ν* is the light frequency, and *E*_g_ is the band gap energy. The bandgap energy was calculated by linear fit extrapolation of a plot of (*αhν*)^2^*vs.* energy (*hv*).

For ATR-FTIR, an FT-IR spectrophotometer (Vertex-70, Bruker Optics®) was used, equipped with an ATR unit (ZnSe crystal) and a liquid N_2_-cooled mercury cadmium telluride (MCT) detector. The extract samples (before and after synthesis) were diluted in ethanol in a 1 : 4 ratio, and the flow rate was adjusted to 5 ml min^−1^. The spectra were recorded using OPUS 6.5 software by performing 128 scans from 4000 to 900 cm^−1^ in absorbance mode with 4 cm^−1^ resolution. The working principle of the set-up is presented in Fig. S1A.†

### 2.4 Catalyst characterization

Crystallographic parameters were examined by XRD (Philips XPERT-PRO) at angles of diffraction (2*θ*) between 15° and 90° using a Cu-Kα radiation source (wavelength *λ* = 1.5406 Å). The measurement was carried out at 40 kV operating voltage and 30 mA current. The results were analyzed by Rietveld refinement using HighScore Plus® (v5.1) connected with ICDD® database. The *d*-spacing (*d*) was calculated based on the following equation (eqn (2)):2
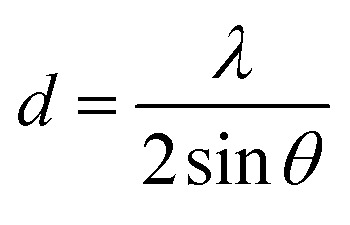
where *λ* is the wavelength of the incident X-ray beam (1.5406 Å), and *θ* is the angle of detector position from the incident X-ray beam. Based on the *d*-spacing values, the average lattice parameter (*a*) was calculated based on the following equation (eqn (3)):3
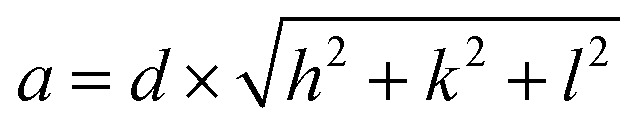
with *h*, *k*, and *l* corresponding to the Miller indices.

N_2_ adsorption–desorption isotherms were recorded at 77 K using an ASAP 2020 instrument (Micromeritics Inc., USA). Before each run, a known mass sample (*ca.* 0.18 g) was heated to 120 °C under vacuum for 2 h. Brunauer–Emmett–Teller (BET) and Barrett–Joyner–Halenda (BJH) models were used to calculate specific surface area and pore size/volume, respectively.

Prior to SEM, specimens were sputter-coated with an 8 nm layer of palladium and gold. The surface morphology was observed using a FEG-250, Quanta instrument applying an accelerating voltage of 5 kV. TEM was performed on a FEI TECNAI F20 field emission microscope equipped with a GATAN GIF Tridiem energy filter and a GATAN Rio16 CMOS camera. The micrographs were analyzed using ImageJ software (1.52a). Selected area electron diffraction (SAED) and EELS were employed to determine the structure and elemental composition in Co_3_O_4_ NMs, respectively.

XPS spectra were acquired at room temperature in a UHV chamber (base pressure <3 × 10^−10^ mbar) equipped with a Specs XR50© high-intensity non-monochromatic Al/Mg dual anode and a Phoibos 100© hemispherical electron energy analyzer with multichannel plate detector. All measurements were performed using the Al anode at 1486.6 eV, at normal emission geometry, and in fixed analyzer transmission with a pass energy of 20 eV. The spectra were calibrated to the C 1s peak at 284.8 eV and analyzed using CasaXPS.

For UATR-FTIR, measurements were done by another Fourier-transform infrared spectrometer (FTIR) (PerkinElmer Universal ATR, PerkinElmer, Waltham, MA, USA) coupled to Frontier Universal Diamond/ZnSe ATR crystal with a pressure arm. Each specimen was suitably pressed against the ATR crystal with the aid of the pressure arm to maintain proper contact between the sample and the ATR crystal. The FTIR spectrometer was operated in the 4000–400 cm^−1^ wavenumber range with 4 cm^−1^ resolution. Absorption intensity *vs.* wavenumber plots were digitally recorded by averaging 64 scans using Spectrum software (PerkinElmer Spectrum 10; PerkinElmer).

H_2_-Temperature Programmed Reduction (H_2_-TPR) experiments were performed in a continuous-flow fixed-bed quartz reactor under atmospheric pressure. For H_2_-TPR, 20 mg of the sample were placed between quartz plugs. After pre-treatment (20 vol% O_2_ in Ar, total flow: 50 ml min^−1^) at 400 °C for 30 min (heating rate 10 °C min^−1^) and cooling to 30 °C in 100 vol% Ar (total flow 50 ml min^−1^), each sample was heated from RT to 800 °C (heating rate of 5 °C min^−1^) in a mixture of 5 vol% H_2_ in Ar (total flow: 50 ml min^−1^). Hydrogen consumption was measured by a quadrupole mass spectrometer (QMS, Prisma Plus QMG 220, Pfeiffer Vacuum) with a MS signal of H_2_ (*m*/*z* = 2) detected online using Quadera software (v.4.40.019).

For O_2_-Temperature Programmed Desorption (O_2_-TPD), 50 mg of a sample was placed between quartz plugs in a continuous-flow reaction system and pre-treated (20 vol% O_2_ in N_2_, total flow: 50 ml min^−1^) at 400 °C for 30 min (heating rate 10 °C min^−1^), followed by cooling down to RT. Then, each sample was heated from RT to 500 °C under vacuum with a heating ramp rate of 10 °C min^−1^. The gas stream was analyzed by a quadruple mass spectrometer (Balzers Prisma QME 200), monitoring the MS signal of O_2_ (*m*/*z* = 32).

### 2.5 Catalytic CO oxidation

Differential scanning calorimetry (DSC) analysis during CO oxidation was performed on Netzsch STA 409 PC Luxx® in an alumina crucible. Before each cycle, samples (10 mg) were pre-treated in 20 vol% O_2_ in He (total flow: 50 ml min^−1^) at 400 °C for 30 min (heating rate 10 °C min^−1^). After cooling down to 25 °C under He, different vol% of CO and O_2_ were introduced under He flow (total flow 20 ml min^−1^) in 10 min intervals: at 10 min CO, 20 min CO + O_2_, 30 min O_2_, 40 min CO + O_2_, and 50 min CO. The scheme of the set-up is presented in Fig. S1B.†

CO oxidation was also conducted in a continuous-flow fixed-bed quartz reactor under atmospheric pressure. Typically, 20 mg of catalyst placed between quartz plugs was loaded into the reactor and pre-treated (20 vol% O_2_ in Ar, total flow: 50 ml min^−1^) at 400 °C for 30 min (heating rate 10 °C min^−1^). The catalyst bed temperature was controlled by a thermocouple. Subsequently, the sample was cooled to 30 °C, and a mixture of 5 vol% CO, 10 vol% O_2_, and 85 vol% Ar (total flow 50 ml min^−1^) was introduced. A quadrupole mass spectrometer (QMS, Prisma Plus QMG 220, Pfeiffer Vacuum) was used to monitor the effluent gas, and the MS signals of CO (*m*/*z* = 28), O_2_ (*m*/*z* = 32), CO_2_ (*m*/*z* = 44) and H_2_O (*m*/*z* = 18) were recorded online using Quadera software (v.4.40.019). The scheme of the set-up is presented in Fig. S1C.†

*In situ* diffuse reflectance infrared Fourier transform spectroscopy (DRIFTS) studies were carried out on a Bruker Vertex 70 spectrometer with a liquid N_2_-cooled MCT detector, a stainless-steel flow cell (Pike) features CaF_2_ windows and an oven. The inlet of the cell was connected to a gas manifold system with calibrated mass flow controllers to adjust the gas mixtures (pre-treatment: 20 vol% O_2_ in Ar, total flow: 50 ml min^−1^, reaction: 5 vol% CO, 10 vol% O_2_ in Ar, total flow: 50 ml min^−1^) and a quadrupole mass spectrometer (QMS, Prisma Plus QMG 220, Pfeiffer Vacuum). Each sample was pre-treated at 400 °C with a temperature ramp of 10 °C min^−1^ and kept at the maximum temperature for 30 min. Next, the sample was cooled to RT (100 vol% Ar), and the gases were switched to reaction conditions. The reaction temperature was increased by 5 °C min^−1^ and kept at the maximum temperature of 300 °C. The overall set-up is presented in Fig. S1B.† DRIFTS spectra were recorded with 4 cm^−1^ resolution using OPUS 6.5 software by averaging 128 scans to achieve a good signal-to-noise ratio. The DRIFTS set-up is presented in Fig. S1D.†

## 3 Results and discussion

### 3.1 Analysis of extracts

**Fig. 2:**
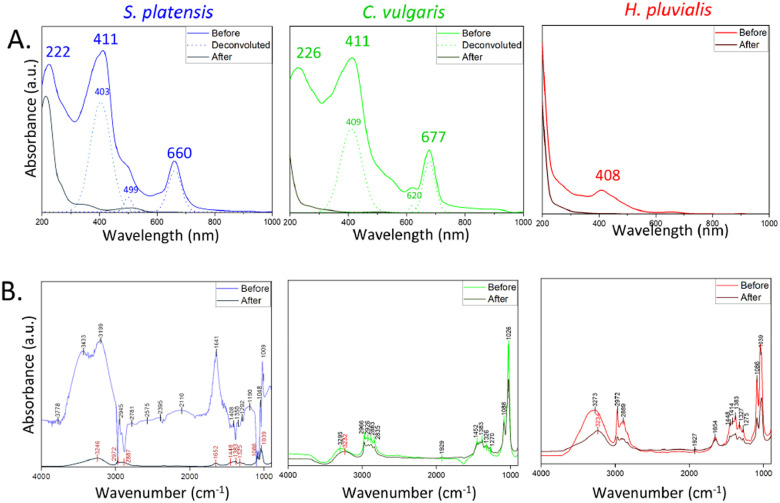
Analysis of the extract before and after synthesis: (A) UV-Vis, (B) ATR-FTIR.

*S. platensis*, *C. vulgaris*, and *H. pluvialis* were investigated to determine differences in their morphology (see optical microscopy images in Fig. S2†) and metabolomic profile.^32,33^*S. platensis* is a well-known source of proteins with less abundant lipid and carbohydrate content. On the contrary, *C. vulgaris* has a decreased protein content and has been used mainly for its high lipid accumulation, while *H. pluvialis* is a source of astaxanthin belonging to carotenoids. Therefore, the effect of the metabolite composition on the synthesis process was evaluated by comparing the extract before and after synthesis by UV-Vis and ATR-FTIR (Fig. 2).

UV-Vis spectra of the extract before and after synthesis (Fig. 1IX and XII) are presented in Fig. 2A. Before synthesis, three major peaks were identified in the *S. platensis* extract: at 222 nm, 411 nm, and 660 nm. The first peak can be assigned to proteins owing to the presence of the carbonyl group in the peptide bond. The same peak was used to determine the presence and isolate various peptides from *S. platensis* by Lu *et al.*^34^ The peaks at 411 nm and 660 nm can be attributed to abundant compounds in the extract, such as carotenoids, chlorophylls, and phycocyanin with overlapping absorbance maxima.^35^ Similar values were observed for *C. vulgaris* extract before synthesis, with peaks at 226 nm, 411 nm, and 677 nm, the redshift probably due to structural differences between molecules belonging to the same group of metabolites.^36^ The *H. pluvialis* extract before synthesis exhibited one major peak at 408 nm characteristic of astaxanthin, usually observed at 450–500 nm. However, the absorbance peak shifted towards 400 nm after water dilution.^37^ After synthesis, all extracts show decreased absorbance, suggesting that the molecules derived from microalgae were indeed involved and employed in the synthesis of Co_3_O_4_ NMs.

The presence of functional groups in the extract before and after synthesis was further investigated by ATR-FTIR. The *S. platensis* extract exhibited a variety of identified functional groups belonging to proteins, lipids, and carbohydrates (Fig. 2B and Table S5†). Comparably, *C. vulgaris* and *H. pluvialis* extracts showed a lower peak intensity for functional groups of proteins and increased intensity of carbonyl groups belonging to carbohydrates (Fig. 2B, Tables S6 and S7†). After synthesis, *S. platensis* extract showed an intensity decrease in all previously identified peaks with visible redshift. In cases of *C. vulgaris* and *H. pluvialis*, the decrease was less significant, and a redshift was observed around 3000 cm^−1^, attributed to the NH group of proteins. In addition, potassium and phosphorous contents were analysed and the values are presented in Table S4.† Altogether, the results confirm a significant involvement of metabolites from the extract, mainly proteins and carbohydrates, in the Co_3_O_4_ NMs synthesis.

Recent studies indicated the potential of extracts to synthesize highly active Co_3_O_4_ NMs. Poonguzhali *et al.* utilized a fresh lemon juice-assisted auto-combustion method to synthesize Co_3_O_4_ NMs as gas sensors.^38^ The response was measured as a change in resistance due to chemical reactivity between the produced oxygen ions and tested gases such as H_2_, CO_2_, LPG, and (CH_3_)_2_CO. The material exhibited a short recovery–response time showing promising gas sensor applications. In a separate study, Khalid *et al.*, tested Co_3_O_4_ NMs prepared from green chili or sunflower seed extracts and compared them with NM synthesized without extract.^39^ The utilization of sunflower seed extract resulted in the synthesis of Co_3_O_4_ NMs with the best photocatalytic and capacitive behaviour among the prepared materials. Although both studies proved the high catalytic activity of Co_3_O_4_ NMs, the extracts used for the synthesis were not analyzed, and therefore the role of the metabolites remained unclear. Herein, the utilization of microalgal metabolites was evaluated to provide insights into the synthesis mechanism. In the next steps, the obtained catalysts were characterized to establish a connection between synthesis, properties, and catalytic activity.

### 3.2 Characterization of Co_3_O_4_ NMs

**Fig. 3:**
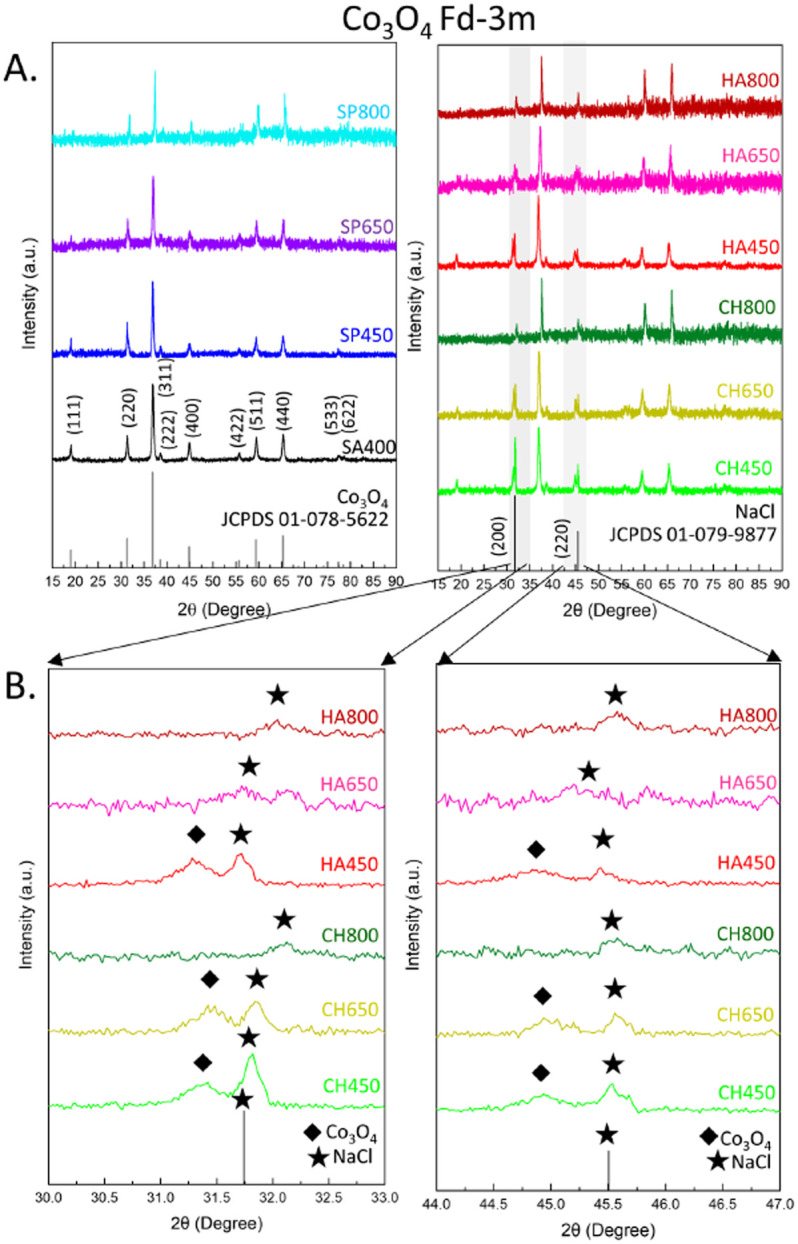
XRD analysis of Co_3_O_4_ NMs: (A) wide angle, (B) selected magnified regions.

The crystal structure of algae-derived cobalt oxide catalysts and the commercial reference were characterized by XRD (Fig. 3). All samples showed a diffraction pattern characteristic of *Fd*3̄*m* cubic spinel Co_3_O_4_ (JCPDS 01-078-5622) with (111), (220), (311), (400), (511), and (440) reflections (Fig. 3A). Co_3_O_4_ NMs synthesized from *C. vulgaris* and *H. pluvialis* extract exhibited additional peaks next to (220) and (400), indicative of NaCl (JCPDS 01-079-9877) formed probably due to the use of NaOH and CoCl_2_·6H_2_O during synthesis.

Another factor that caused changes in the pattern was the calcination temperature which, consistent with literature, shifted all peak positions to higher values due to lattice contraction^40^ (Tables S8–S11†), indicating a more strained crystal lattice. A small decrease was observed for *S. platensis* and *C. vulgaris* between 450 to 650 °C, while a more prominent decrease occurred for other Co_3_O_4_ NMs (Table 1 and Fig. S4B†). A similar tendency was observed for Co_3_O_4_ NMs synthesized *via* a chemical method and calcined at 400, 600, and 800 °C.^41^ The applied thermal energy contributed to a more ordered ion arrangement, decreasing the unit cell size (lattice contraction).

**Table 1:** Structural properties of the different Co_3_O_4_ NMs

Sample	Crystallite size (nm)	Lattice parameter *a* = *b* = *c* (Å)	BJH adsorption cumulative pores volume (cm^3^ g^−1^)	BJH average pore width (nm)	BET surface area (m^2^ g^−1^)
SA400	21.9	8.067	0.266	26.8	43.7
SP450	26.1	8.068	0.029	16.1	6.9
SP650	24.1	8.057	0.024	18.5	6.1
SP800	28.6	7.980	0.012	38.1	3.4
CH450	16.4	8.058	0.068	25.6	13.3
CH650	24.0	8.054	0.074	37.3	11.3
CH800	40.3	7.951	0.019	28.6	4.6
HA450	18.4	8.078	0.171	24.4	29.2
HA650	23.1	8.014	0.141	39.2	19.5
HA800	42.2	7.958	0.015	14.7	5.0

Structural changes were observed for HA650, for which (111) was absent, as well as CH800 and HA800, for which (111), (220), and (400) were not present (Fig. 3B). In addition, crystallite sizes increased upon calcination, indicating sintering of Co_3_O_4_ NMs: slight changes for Co_3_O_4_ NMs from *S. platensis*, but more pronounced growth for Co_3_O_4_ NMs from *C. vulgaris* and *H. pluvialis* (Table 1 and Fig. S4A†).

N_2_ physisorption isotherms were acquired to determine the textural properties of Co_3_O_4_ NMs. According to the IUPAC classification, the adsorption–desorption isotherms belong to type IV isotherms with an H1 hysteresis loop suggesting the presence of meso- and macro-pores (Fig. S5†).^42^ The pore distribution graphs, also shown in Fig. S6,† reveal a narrow spread of 2.5–4 nm width for all samples. In addition, SA400 Co_3_O_4_ NMs show a bell-shaped pore distribution around 5–80 nm. The high porosity correlates with a large surface area of SA400 Co_3_O_4_ NMs (43.7 m^2^ g^−1^) and huge pores volume (Table 1). However, even though SA400 Co_3_O_4_ NMs, CH450 Co_3_O_4_ NMs, and HA450 Co_3_O_4_ NMs show similar average pore width, their pore volume varies, suggesting pore blocking in the catalysts synthesized using microalgae. The porosity change with increasing temperature has been reported before.^43^ The calcination temperature was found to increase Co atom migration that leads to bigger Co_3_O_4_ crystallites which, in turn, causes structural strain in the material. This results in a shrinkage of the formed porous structures which finally collapse into quasi-spherical particles. Moreover, the gases formed during organic residue decomposition can further destroy structural integrity, or they may lead to carbon deposition decreasing the catalytic activity. These findings have been confirmed in the current study. Increasing the calcination temperature decreased porosity, pore volume, and surface area, with the most significant surface area decrease for Co_3_O_4_ NMs obtained from *H. pluvialis* extract (from 29.2 to 5 m^2^ g^−1^). A similar effect was observed for Co_3_O_4_ NMs synthesized using a metal–organic framework, with the surface area decreasing from 120.9 m^2^ g^−1^ to 22.6 m^2^ g^−1^ upon calcination temperature increase from 300 to 400 °C, likely due to sintering.^43^ The effect of calcination temperature on the surface area is presented in Fig. S4C.†

**Fig. 4:**
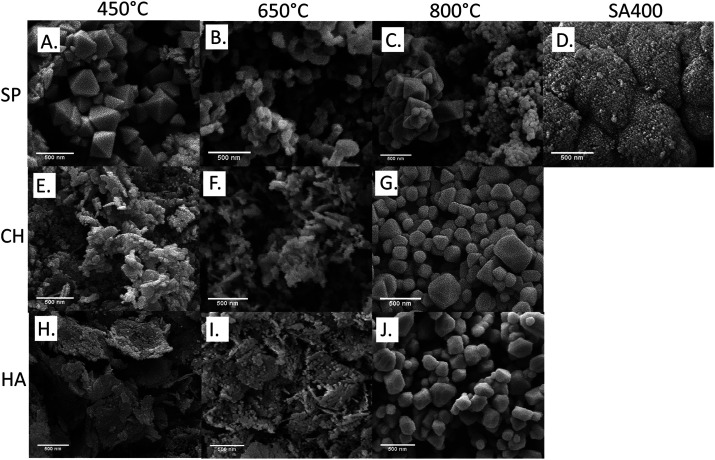
SEM analysis of Co_3_O_4_ NMs: (A) SP450, (B) SP650, (C) SP800, (D) SA400, (E) CH450, (F) CH650, (G) CH800, (H) HA450, (I) HA650, (J) HA800.

The morphologies of the synthesized and commercial Co_3_O_4_ NMs were examined by SEM. As presented in Fig. 4, SA400 Co_3_O_4_ NMs were spherical or quasi-spherical agglomerates with high porosity confirming the BET findings. Octahedral shape was observed for SP450 Co_3_O_4_ NMs with more abundant hollow spherical agglomerates upon increasing calcination temperature. Nanosheet morphologies were observed for CH450 and HA450 Co_3_O_4_ NMs with similar tendency regarding spherical form upon rising calcination temperature, as described previously.^43^ High resolution TEM images showed lattice fringes corresponding to (111) crystalline planes (Fig. S6A, C, E and G†). The obtained SAED patterns (Fig. S6B, D, F and H†) of the materials showed characteristic diffraction rings, which can be attributed to (111), (220), (311), (400), (511), and (440), in line with the XRD findings.

The local valence state of Co_3_O_4_ NMs was studied in the TEM using the EELS technique (Fig. S7†). The O K-edge displays peaks a, b, and c in the 533–553 eV range. Peak a, referred to as the pre-edge peak, is ascribed to O 2p unoccupied states hybridization with the Co 3d orbital, while peaks b and c originate from O 2p state hybridization with the Co 4sp band.^44^ The increased pre-edge peak intensity compared with peak b is considered as a fingerprint of Co_3_O_4_ due to the high number of unoccupied Co 3d states.^45^ Moreover, the decreased electron counts of peaks a and b are correlated with oxygen vacancies in Co_3_O_4_ NMs,^46,47^ with rich oxygen vacancy indicated for SA400 Co_3_O_4_ NMs, followed by CH450, HA450, and SP450 Co_3_O_4_ NMs. The EELS spectra also display Co L_3_ and L_2_ edges at 780–800 eV, stemming from 2p_3/2_ and 2p_1/2_ core–shell electron transition into 3d orbitals in a pattern typical for Co_3_O_4_.^44^

**Fig. 5:**
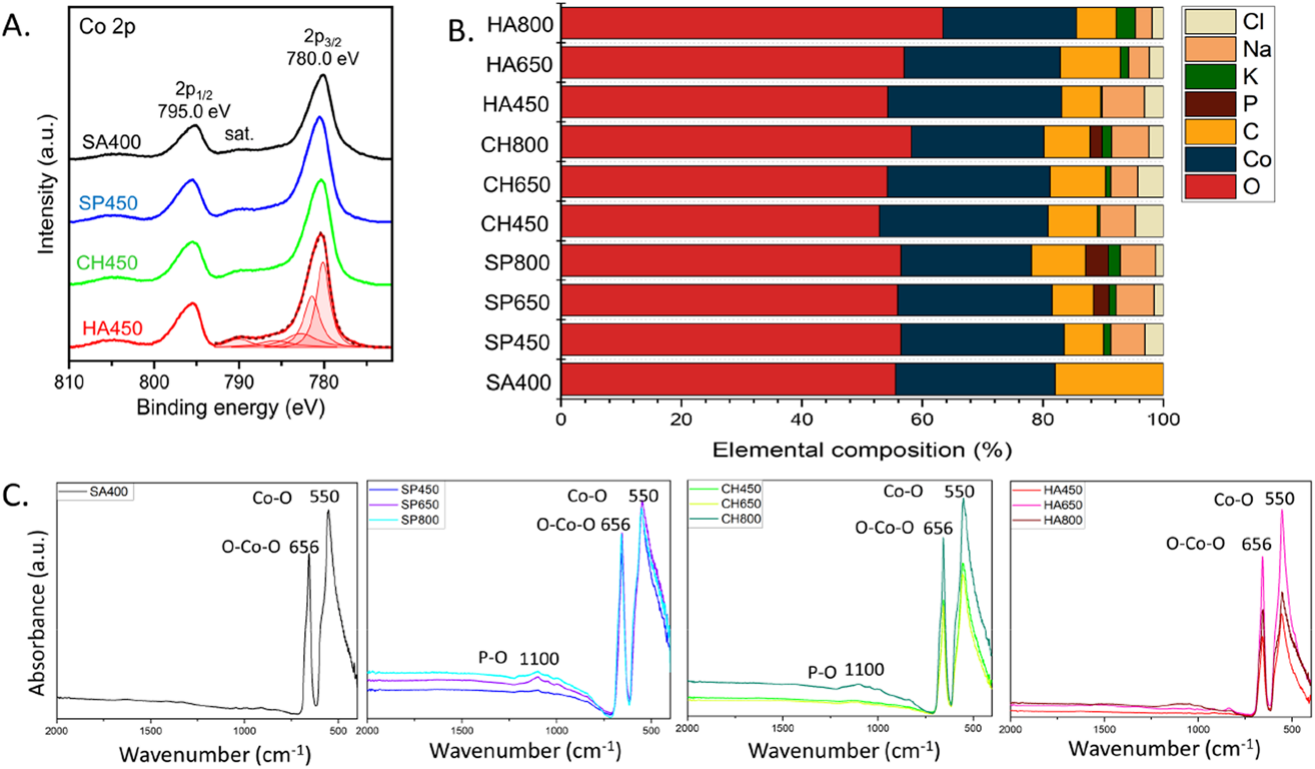
Surface composition of Co_3_O_4_ NMs: (A) XPS Co 2p spectra, (B) elemental composition based on XPS analysis, (C) UATR-FTIR spectra.

The catalyst surface composition was also examined by XPS, which provided evidence of the Co_3_O_4_ phase (Fig. 5A), confirming XRD and EELS. The region exhibits a doublet at 280.0 and 295 eV of Co 2p_3/2_ and Co 2p_1/2_ respectively, as well as satellite structures. While the binding energy of oxidized Co species are near identical, the peak shape clearly indicates Co_3_O_4_.^48,49^ Further, the Co 2p_3/2_ envelope matches the data perfectly, when fitted according to procedures for Co_3_O_4_ (using constraints for peak areas and positions), as shown for HA450. The same holds true for the analogous peaks of SP450, CH450 and SA400.

The relative surface composition, derived from XPS, is displayed in Fig. 5B. The samples have similar oxygen content with a slight increase upon rising calcination temperature. The Co_3_O_4_ NMs synthesized using microalgae extract also showed the presence of sodium (from NaOH) and chlorine (from cobalt salt) added during the synthesis process, as also observed by XRD. In addition, Co_3_O_4_ NMs synthesized using microalgal extract contained potassium, whose percentage increased with calcination temperature, and phosphorus that was observed only in SP650, SP800, and CH800 Co_3_O_4_ NMs. Both potassium and phosphorus originated from the microalgae extract (Table S4†), which once more demonstrates the involvement of metabolites during synthesis. The variable potassium, phosphorus, carbon, and oxygen contents suggest surface segregation, which may also cause the differences in the Co_3_O_4_ NMs crystallographic parameters (Table 1).

The XPS results were confirmed by UATR-FTIR analysis of Co_3_O_4_ NMs, which also revealed the presence of phosphorous *via* a phosphorous–oxygen stretching vibration at 1100 cm^−1^ (ref. 50) in SP650, SP800, and CH800 Co_3_O_4_ NMs (Fig. 5C). All UATR-FTIR spectra showed peaks at 550 cm^−1^ belonging to Co–O stretching and 656 cm^−1^ corresponding to O–Co–O bridging vibration due to Co–O linkage.^51^ Moreover, no organic ligands were detected on the surface (Fig. S8†).

### 3.3 Oxygen availability of Co_3_O_4_ NMs

The address the reducibility/stability of the cobalt catalysts, H_2_-TPR was carried out, as presented in Fig. S9.† Typically, Co_3_O_4_ NMs exhibit two peaks corresponding to reduction of Co^3+^ to Co^2+^ and Co^2+^ to Co^0^, which may partly overlap. A lower temperature of H_2_ consumption indicates weaker Co–O bond strength, which should lead to better accessible oxygen species and thus higher oxidation activity.^52^

The lowest TPR temperatures of both peaks were observed for SA400 Co_3_O_4_ NMs, followed by HA450, CH450, and SP450 Co_3_O_4_ NMs. The lower temperature for HA450 than CH450 Co_3_O_4_ NMs shows the potential for oxidation performance, which could be hindered by other factors, such as the presence of sodium and chlorine. Increasing the calcination temperature shifted the peaks to higher TPR temperature, which should be a descriptor of lower activity. For HA650 and HA800 Co_3_O_4_ NMs, only one TPR peak was observed anymore, likely due to calcination-induced changes in the structure. The reducibility/stability of the cobalt catalysts was examined by H_2_-TPR, as presented in Fig. S9.† The detected peaks are also presented in Table S12.†

**Fig. 6:**
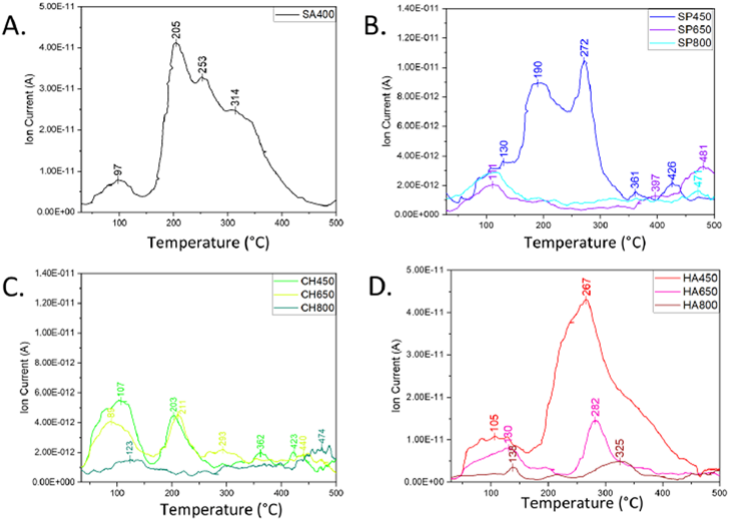
O_2_-TPD of Co_3_O_4_ NMs: (A) SP400, (B) SP450, SP650, SP800, (C) CH450, CH650, CH800, (D) HA450, HA6550, HA800.

The availability of oxygen species was directly investigated by O_2_-TPD, *i.e.* the desorption of different oxygen species from the catalysts' surface (Fig. 6). Three types of oxygen species were detected in the spectra and, according to the literature,^53^ they can be assigned depending on their temperature range: physisorbed molecular and/or chemisorbed dissociated surface oxygen species desorbing at 50–150 °C, surface lattice oxygen desorbing at 150–450 °C rather easily forming oxygen vacancies, and bulk oxygen desorbing at 450–500 °C.^53^ The most intense signal was observed for SA400 Co_3_O_4_ NMs, mostly as surface lattice oxygen, in line with efficient oxidation performance following the Mars–van-Krevelen (MvK) mechanism. Abundant surface-active oxygens were also observed for HA450 and SP450 Co_3_O_4_ NMs. On the contrary, CH450 Co_3_O_4_ NMs showed an increased amount of molecular oxygen, which should lead to better catalytic performance than the other Co_3_O_4_ NMs synthesized from microalgal extract. Moreover, increasing the calcination temperature decreased the amount of desorbing oxygen, thus decreasing the Co_3_O_4_ NMs CO oxidation ability.

### 3.4 CO oxidation on Co_3_O_4_ NMs

**Fig. 7:**
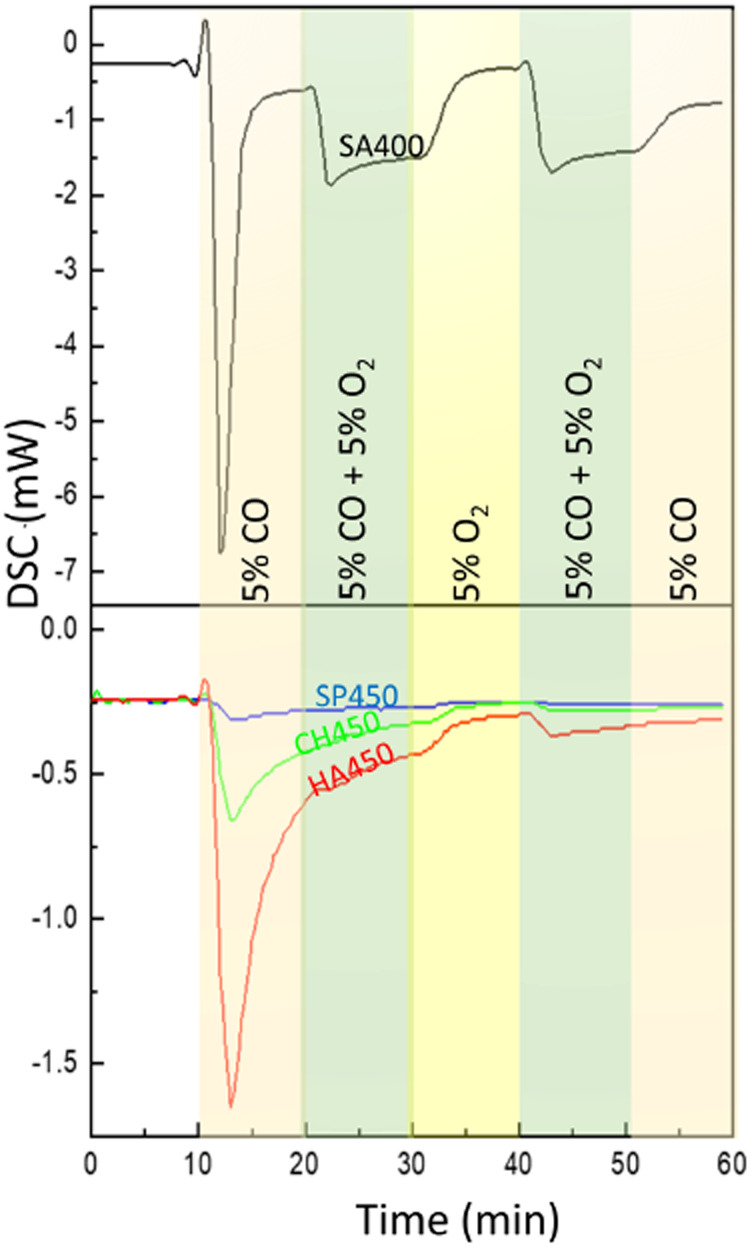
*In situ* DSC analysis of Co_3_O_4_ NMs: SA400, SP450, CH450, and HA450.

To evaluate CO oxidation on the Co_3_O_4_ NMs, *in situ* DSC was applied (Fig. 7). Upon CO exposure, several processes can take place including adsorption, carbonate and CO_2_ formation.^8,54^ Both CO adsorption and oxidation are exothermic and can occur in parallel. The energy released was calculated based on the measured peak areas, as presented in Table S13.† For CO exposure, the strongest exothermicity with −14.4 J g^−1^ was observed for SA400 Co_3_O_4_ NMs, with significantly reduced values of −0.4, −2.4, and −6.5 J g^−1^ for SP450, CH450, and HA450 Co_3_O_4_ NMs, respectively. For CO_2_ formation, CO interacts with active Co sites and neighboring oxygen. The generated oxygen vacancy can be replenished by gas phase oxygen, but CO adsorption may also lead to dissociation and carbon deposition (which may be re-oxidized upon oxygen exposure). The strongest exothermicity for SA400 Co_3_O_4_ NMs is connected with its high activity, whereas the differences to other samples might originate from the formation of different carbonate species on the surface.

Co-dosing molecular oxygen then led to much smaller heat release (Fig. 7). This difference may be due to active oxygen species that were only present after pretreatment in oxygen.^8^ Even after a 10 min treatment with (pure) oxygen, the heat release remained reduced, *i.e.* the surface oxygen species were not replenished. A decrease in exothermicity was also observed for SA400 Co_3_O_4_ NMs in a following experiment with different gas composition (Fig. S10 and Table S14†). The results suggest a complex network of different interactions due to CO and O_2_ interacting with different sites, as discussed previously.^8,26^

**Fig. 8:**
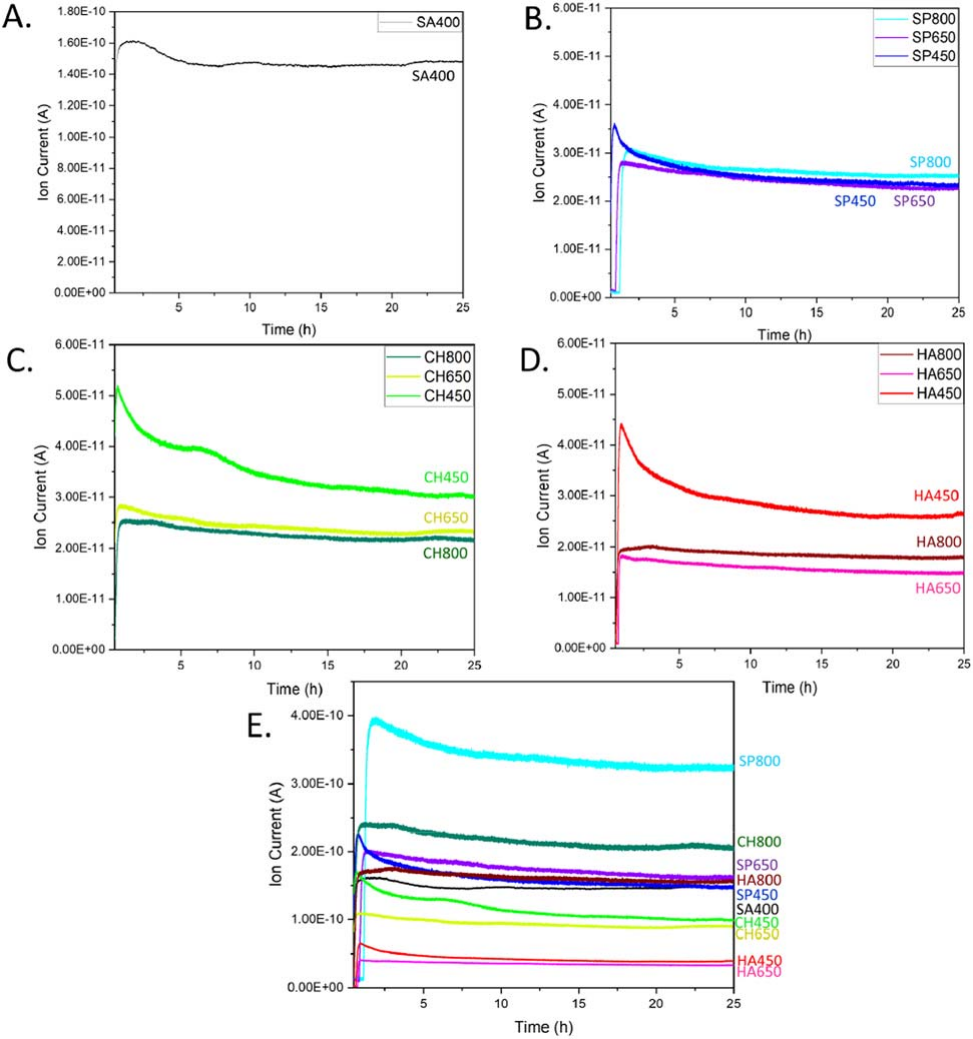
CO oxidation at room temperature over Co_3_O_4_ NMs: MS spectra of CO_2_ for (A) SA400, (B) SP, (C) CH, (D) HA. (E) CO_2_ signal normalized per surface area of SA400.

The various catalysts were then tested for CO oxidation in an atmospheric flow reactor, with results shown in Fig. 8. The CO oxidation activity of algae-derived Co_3_O_4_ NMs extract is described here for the first time along with a comparison to commercial Co_3_O_4_. The most intense CO_2_ signal, corresponding to the highest catalytic activity, was observed for SA400 Co_3_O_4_ NMs, followed by CH450, HA450, and SP450 Co_3_O_4_ NMs. This order correlates well with the one of oxygen vacancy content revealed by EELS. Among the tested microalgae, after calcination at 800 °C, Co_3_O_4_ NMs synthesized using *S. platensis* extract were the most active (SP800, Fig. S11†). For calcination at lower temperature, the highest activity was observed for the Co_3_O_4_ NMs synthesized using *C. vulgaris* extract (CH450, CH650).

Increasing the calcination temperature rather decreased the catalytic activity of Co_3_O_4_ NMs synthesized from microalgae, which may be related to a decrease in surface area (Table 1). However, it increased the signal stability, with an activity decrease of only 11–19% for Co_3_O_4_ NMs calcined at 800 °C and 35–41% for Co_3_O_4_ NMs calcined at 450 °C (Fig. S12†).

For better and direct comparison, the catalytic activity was also normalized by the corresponding catalyst surface area, including SA400 as reference (Fig. 8E), revealing higher (normalized) catalytic activity of SP800, CH800, and SP650 Co_3_O_4_ NMs, which contained phosphorous, as indicated by XPS. The effect of phosphorus has been studied before, mainly as a dopant in Co_3_O_4_ NMs. During peroxymonosulfate (PMS) activation, due to the presence of phosphorus, more active cobalt sites were exposed, thereby exhibiting higher affinity and easier electron transfer for PMS, while weakening the O–O bond for the rapid generation of oxygen radicals.^55^

On the oxide catalysts of the current study, CO oxidation mainly follows a Mars–van-Krevelen (MvK) mechanism which involves reactant adsorption, lattice oxygen activation, reaction with active lattice oxygen, and CO_2_ desorption, paralleled by replenishment of oxygen vacancies by gas-phase oxygen.^8,56^ Although reaction of CO and chemisorbed oxygen cannot be fully excluded (Langmuir–Hinshelwood mechanism),^26^ an increased oxygen mobility induced by phosphorous should improve the catalytic activity. The promoting effect of phosphorous persists despite the presence of sodium and chlorine, which were reported to reduce oxygen mobility, thus rather poisoning catalytic oxidation.^30,31^ However, a lower sodium and chlorine content in HA650 Co_3_O_4_ NMs than in HA450 Co_3_O_4_ NMs did not increase the activity of the first. Nevertheless, HA800 Co_3_O_4_ NMs showed much better performance, probably due to higher potassium content. Potassium in the Co_3_O_4_ NMs lattice was assigned to promote oxygen activation by facilitating CO_2_ desorption, resulting in a more facile CO oxidation process.^28^ Accelerated charge transfer was also observed for Co_3_O_4_ anchored on nitrogen-doped carbon nanotubes, upon oxygen vacancies mobility in Co_3_O_4_, inducing active species Co–O–P.^52^ Moreover, the resulting lattice dislocations were examined for their enhanced adsorption and promoting polysulfide conversion activity.^29^ So far, the effects of phosphorous, potassium, chlorine, and sodium have been described, but only when the elements were introduced separately to Co_3_O_4_ NMs, whereas their simultaneous effect on CO oxidation had not been tested.

The catalytic activity was also correlated with the bandgap energy (Fig. S13 and Table S15†), representing the minimum energy required to excite an electron from the valence band to the conduction band. The lowest values were noted for SP800, CH800, and SP650 Co_3_O_4_ NMs, following the order of activity as depicted in Fig. 8E. The values belong to the charge transfer range between heterovalent cobalt ions, which may explain their electron mobility and lattice oxygen activity.^57^

**Fig. 9:**
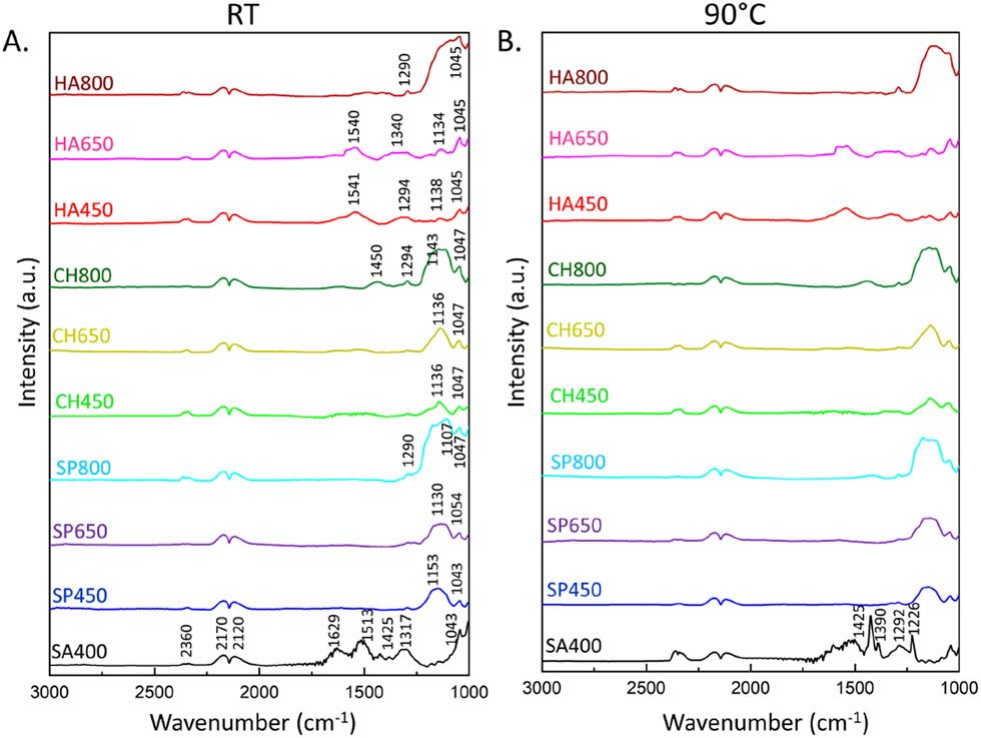
*In situ* DRIFTS spectra of CO oxidation on Co_3_O_4_ NMs: (A) at room temperature (RT) after 25 minutes of the reaction, (B) at 90 °C after 13 minutes of the reaction.

The catalytic reaction was also monitored by *in situ* DRIFTS, detecting species absorbed on the catalysts (Fig. 9, S14 and S15†). During CO oxidation, the IR spectra revealed gas phase bands of the product CO_2_ at 2360 cm^−1^, as well as peaks of gas phase CO at 2170 and 2120 cm^−1^.^58^ The peaks in the 1000–1700 cm^−1^ region correspond to various vibrations of adsorbed carbonates, likely just acting as spectators.^26,59^ The SA400 Co_3_O_4_ NMs showed formation of bridging carbonates at 1629 cm^−1^, bidentate at 1513 cm^−1^, and monodentate at 1425, 1317, and 1043 cm^−1^, while Co_3_O_4_ NMs synthesized using microalgae extract showed mostly monodentate (1541, 1540, 1450, 1340, 1294, 1290, 1051, 1054, 1050, 1047, 1045, 1043) and bridged carbonates (1153, 1143, 1138, 1136, 1134, 1130, 1111, 1107).^26^ The higher intensity of carbonate formation in SA400 Co_3_O_4_ NMs correlates with its higher CO_2_ production activity, while other Co_3_O_4_ NMs showed less pronounced performance with a tendency towards monodentate formation upon higher calcination temperature. This trend suggests a simpler binding geometry compared to bidentate or bridging carbonates, potentially resulting in lower structural complexity of carbonates on the catalyst surface. The carbonates were formed as soon as Co_3_O_4_ MNs were exposed to the reaction mixture, but their build-up was suppressed as the reaction progressed. Therefore, the formation of carbonates is likely limited by the adsorption sites on the catalyst surface. In addition, Co_3_O_4_ NMs synthesized using the same microalgae extract but subjected to different calcination temperatures show a variation in the detected IR spectra reflecting differences in the surface properties which could have an impact on the reaction pathway and the overall catalytic activity. Although the carbonates are not considered active participants during CO oxidation and usually act as spectators, they can behave as a physical barrier on the surface of the catalyst. The presence of surface carbonates may physically hinder the adsorption of CO or oxygen molecules onto the active sites of the catalyst, influencing the overall catalytic activity.^8^ Carbonates may cover or block active sites on the catalyst surface where CO or oxygen molecules would typically adsorb.

The properties of the formed carbonates were further studied by thermal exposure. When the temperature was increased to 90 °C on SA400 Co_3_O_4_ NMs (Fig. 9B), the carbonate species decreased, whereas more gaseous CO_2_ was visible at 2360 cm^−1^. In the case of microalgae-derived Co_3_O_4_ NMs, the carbonate species rather remained adsorbed on the surface, suggesting higher thermal stability. A more stable carbonate intermediate suggests a catalyst that can endure prolonged exposure to high temperatures without undergoing significant structural changes, impacting both environmental and industrial applications.

Although the surface area of Co_3_O_4_ NMs obtained *via* a biological method was smaller than that of commercial Co_3_O_4_ NMs, their normalized catalytic activity showed the potential to improve CO oxidation due to microalgal components such as phosphorous or potassium. As a result, the environmentally harmful effect of CO emission to the atmosphere can be mitigated using sustainable catalyst preparation methods. In addition, insights gained from CO oxidation, which serves as a well-known benchmark reaction, may contribute to a better understanding of oxide-gas interactions and oxidative removal of other hazardous pollutants.

## 4 Conclusions

The present study exploited how extracts from three different microalgae can be employed for the synthesis of various Co_3_O_4_ NMs, further modified by different calcination temperatures, and using commercial spinel Co_3_O_4_ as reference. The focus was placed on the relationship between the structure, morphology, composition, reducibility, oxygen species and CO oxidation activity of the various Co_3_O_4_ NMs. The catalysts' structure/composition were characterized by XRD, SEM/TEM/EELS, XPS, their reducibility by H_2_-TPR, and oxygen availability by O_2_-TPD. Higher calcination temperature caused surface segregation of phosphorous and potassium, originating from the extract, which seems to promote the Co_3_O_4_ NMs in CO oxidation. Although commercial Co_3_O_4_ NMs exhibited the highest activity, when normalized to surface area, the activity of Co_3_O_4_ NMs derived from microalgae and calcined at high temperature was superior. The presence of phosphorous and potassium enhanced the catalytic activity, despite a previously reported poisoning effect of sodium and chlorine, which were also present as synthesis residues. During CO oxidation, *in situ* DSC and *in situ* DRIFTS revealed interactions between reactants and Co_3_O_4_ NMs, but also complexity related to pretreatment effects. The low surface area of Co_3_O_4_ NMs synthesized from microalgae is currently a limiting factor, which can be mitigated by future studies using modified synthesis techniques yielding higher surface area, creating highly active Co_3_O_4_ NMs based on biological extracts. Overall, the present study highlights the potential of microalgae to synthesize nanomaterials along environmentally-friendly routes, which can be used in automotive catalytic converters for reducing CO emissions, petrochemical refineries to comply with environmental regulations, and can play a key role in fuel cells by preventing carbon monoxide poisoning while ensuring efficient operation.

## Author contributions

A. S.: investigation; methodology; data curation; software; formal analysis; validation; conceptualization; visualization; writing – original draft; writing – review & editing, N. Y.: investigation; methodology; data curation, T. W.: investigation; writing – review & editing, M. S. P.: resources; investigation, A. C.: investigation; methodology; writing – review & editing, R. O.: resources; writing – review & editing, G. C.: funding acquisition; resources; project administration; writing – review & editing, G. R.: supervision; conceptualization; methodology; resources; validation; project administration; writing – review & editing.

## Conflicts of interest

There are no conflicts to declare.

## Supplementary Material

RA-014-D4RA00343H-s001
