# Supplementary material for: Microalgae-derived Co3O4 nanomaterials for catalytic CO oxidation
Source: RSC Adv. 2024 Feb 5;14(7):4575–86. doi: 10.1039/d4ra00343h (PMC10839636; doi:10.1039/d4ra00343h)
Supplement: RA-014-D4RA00343H-s001 [file RA-014-D4RA00343H-s001.pdf]

Electronic Supplementary Material (ESI) for RSC Advances.
This journal is © The Royal Society of Chemistry 2024

Supplementary information

Microalgae-derived Co_3O_4 nanomaterials for catalytic CO oxidation

Agnieszka Sidorowicz, [a] Nevzat Yigit,[b] Thomas Wicht,[b] Michael Stöger-Pollach,[c] Alessandro Concas,[a] Roberto Orrù,[a] Giacomo Cao,[a,*] and Günther Rupprechter[b,*]

[a]Interdepartmental Centre of Environmental Engineering and Sciences, University of Cagliari, 09123 Cagliari, Italy

[b]Institute of Materials Chemistry, TU Wien, Getreidemarkt 9/ BC, 1060 Vienna, Austria

[c]University Service Center for Transmission Electron Microscopy, TU Wien, Wiedner Hauptstr. 8-10, 1040 Vienna, Austria

Table of contents

Table S1 Used media composition for Spirulina platensis cultivation. 3
Table S2 Used media composition for Chlorella vulgaris cultivation. 4
Table S3 Used media composition for Haematococcus pluvialis cultivation. 4
Figure S1 Experimental set-up (A) ATR-FTIR, (B) DSC, (C) CO oxidation, (D) DRIFTS. 5
Figure S2 Optical microscopy images of the used microalgae (A) Spirulina platensis, (B) Chlorella vulgaris, (C) Haematococcus pluvialis. 5
Table S4 Elemental analysis of the used microalgae extract. 5
Table S5 FTIR values of Spirulina platensis extract. 6
Table S6 FTIR values of Chlorella vulgaris extract. 7
Table S7 FTIR values of Haematococcus pluvialis extract. 8
Figure S3 XRD analysis of Co_3O_4 NMs: (A) wide angle, (B) selected magnified angle regions. 9
Table S8 D-spacing values of SA400 Co_3O_4 NMs. 9
Table S9 D-spacing values for SP450, SP650, and SP800 Co_3O_4 NMs. 10
Table S10 D-spacing values for CH450, CH650, and CH800 Co_3O_4 NMs. 10
Table S11 D-spacing values for HA450, HA650, and HA800 Co_3O_4 NMs. 11
Figure S4 Influence of calcination temperature (A) crystallite size, (B) lattice parameter and volume, (C) surface area. 11
Figure S5 BET isotherms for commercial and synthesized Co_3O_4 NMs. 12
Figure S6 TEM and SAED studies of Co_3O_4 NMs: (A-B) SA400, (C-D) SP450, (E-F) CH450, (G-H) HA450. 12
Figure S7 EELS spectra of the commercial and synthesized Co_3O_4 NMs (A) SA, (B) SP450, (C) CH450, (D) HA450. 13
Figure S8 UATR-FTIR spectra of Co_3O_4 NMs. 13
Figure S9 H_2-TPR of Co_3O_4 NMs (A) SP400, (B) SP450, SP650, SP800, (C) CH450, CH650, CH800, (D) HA450, HA6550, HA800. 14
Table S12 Detected peaks in H_2-TPR and O_2-TPD (°C) for Co_3O_4 NMs. 14
Table S13 DSC activation energy values (J/g) for SA, SP450, CH450, and HA450 Co_3O_4 NMs. 15
Figure S10 DSC measurements for SA400 Co_3O_4 NMs. 15
Table S14 DCS activation energy values (J/g) for SA400 Co_3O_4 NMs. 16
Figure S11 CO oxidation activity comparison between Co_3O_4 NMs calcined at different temperatures: (A) SP450, CH450, HA450, (B) SP650, CH650, HA650, (C) SP800, CH800, HA800. 16
Figure S12 CO oxidation activity decrease with time of Co_3O_4 NMs: (A), SA400, (B) SP, (C) CH, (D) HA. 17
Figure S13 Tauc plots for commercial and synthesized Co_3O_4 NMs. 17
Table S15 Bandgap values of Co_3O_4 NMs. 19
Figure S14 DRIFTS spectra of SA400, SP450, CH450, and HA450 (A) CO oxidation activity over time (carbonate formation), (B) CO oxidation activity upon heating (carbonate stability). 19
Figure S15 DRIFTS spectra of SP650, SP800, CH650, CH800, HA450, and HA800 (A) CO oxidation activity over time (carbonate formation), (B) CO oxidation activity upon heating (carbonate stability). 20

Table S1 Used media composition for *Spirulina platensis* cultivation.

Components in Modified Zarrouk Medium	Concentration (mg/L)
$NaHCO_3$	7000.0
$NaNO_3$	3000.0
K_2HPO_4	800.0
K_2SO_4	400.0
$MgSO_4$	240.0
$CaCl_2 \cdot 2\ H_2O$	40.0
NaCl	1000.0
Na-EDTA$\cdot$2 H_2O	100.0
KOH	1000.0
$FeSO_4 \cdot 7\ H_2O$	10.0
EDTA	0.05000
H_3BO_3	0.01140
ZnSO4$\cdot$7 H_2O	0.02200
$MnCl_2 \cdot 4\ H_2O$	0.00506
$CoCl_2 \cdot 6\ H_2O$	0.00161
$CuSO_4 \cdot 5\ H_2O$	0.00157
$(NH_4)_6Mo_7O_{24} \cdot 4\ H_2O$	0.00110

Table S2 Used media composition for *Chlorella vulgaris* cultivation.

Components in BBM Medium	Concentration (mg/L)
$NaNO_3$	250.0
K_2HPO_4	75.0
KH_2PO_4	175.0
$MgSO_4 \cdot 7\ H_2O$	75.0
$CaCl_2 \cdot 2\ H_2O$	25.0
NaCl	25.0
EDTA	63.6
KOH	31.0
$FeSO_4 \cdot 7\ H_2O$ in H_2SO_4 (10%)	5.0
ZnSO4·7 H_2O	8.82
CuSO4·5 H_2O	1.57
MnCl2·4 H_2O	1.44
MoO_3	0.71
$Co(NO_3)_2 \cdot 6\ H_2O$	0.49

Table S3 Used media composition for *Haematococcus pluvialis* cultivation.

Components in Modified OHM Medium	Concentration (mg/L)
KNO_3	410.0
K_2HPO_4	30.0
$MgSO_4 \cdot 7\ H_2O$	246.5
$CaCl_2 \cdot 2\ H_2O$	111.0
$FeSO_4 \cdot 7\ H_2O$	3.0
$MnCl_2$·4 H2O	0.989
$(NH_4)_6Mo_7O_{24} \cdot 4\ H_2O$	0.120
$CuSO_4 \cdot 5\ H_2O$	0.012
$CoCl_2 \cdot 6\ H_2O$	0.011
Se	0.005

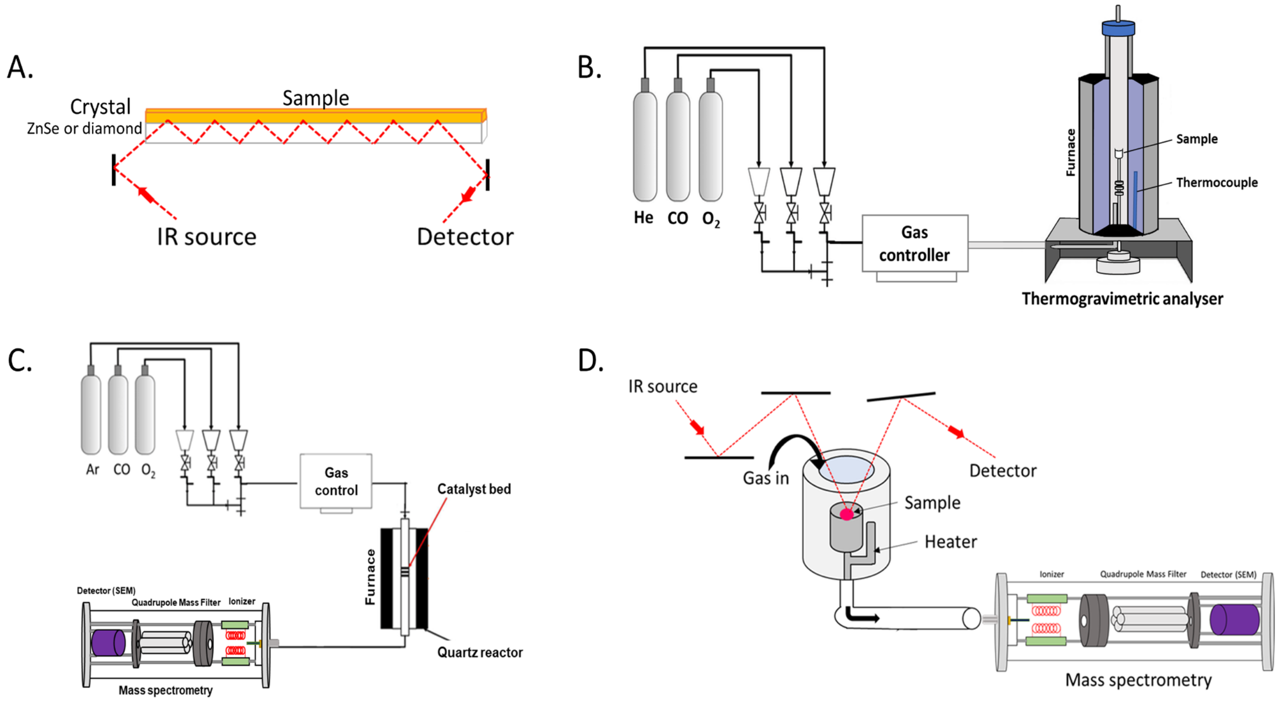

Figure S1 Experimental set-up (A) ATR-FTIR, (B) DSC, (C) CO oxidation, (D) DRIFTS.

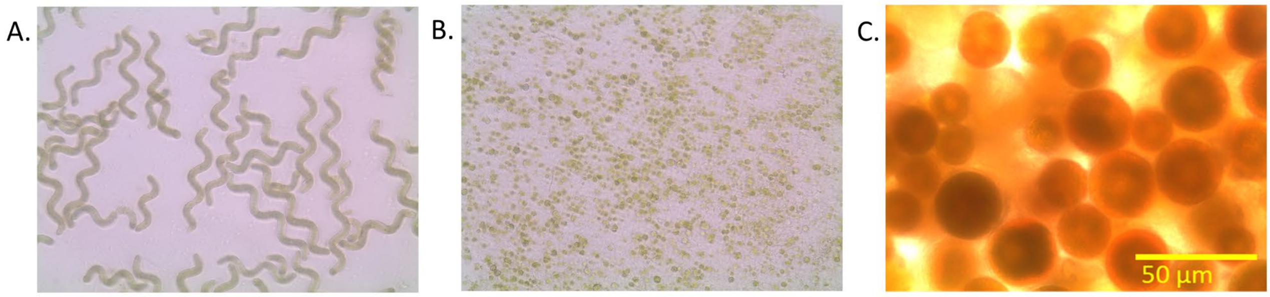

Figure S2 Optical microscopy images of the used microalgae: (A) *Spirulina platensis*, (B) *Chlorella vulgaris*, (C) *Haematococcus pluvialis.*

Table S4 Elemental analysis of the used microalgae extract.

	Phosphorous content (mg/L)	Potassium content (mg/L)
Spirulina platensis	1.070	100.0
Chlorella vulgaris	1.595	90.0
Haematococcus pluvialis	0.405	19.5

Table S5 FTIR values of *Spirulina platensis* extract.

Spirulina platensis [1–3]		
Wavenumber	Functional group	Characteristics
3778, 3433, 3246, 3199	O-H and N-H	OH stretching vibrations of polysaccharides, proteins and water<br>NH stretching vibrations of proteins
2972	CH_3	Asymmetric stretching vibrations of C-H in CH_3 group
2945	CH_2	Asymmetric stretching vibrations of C-H in CH_2 group
2887	CH_3	Symmetric stretching vibrations of C-H in CH_3 group
2781	C-H	Stretching vibrations of cyclic aliphatic hydrocations
2575, 2395, 2110	C=C and C≡C	Stretching vibrations of conjugated systems
1652, 1641	C=O	Amide I band
1448, 1383	CH_3 and CH_2	C-H bending vibrations of lipids
1408	CH_3 and CH_2	C-H bending vibrations
1350, 1325	C-N	Amide III band
1292	C-N	Stretching vibrations of CO-NH
1190	C-O	Stretching vibrations of C-O-C in polysaccharides
1086	C-O and PO^{2-}	Stretching vibrations in carbohydrates and stretching vibrations of phosphate group
1048, 1039	C-O	Stretching vibrations of C-O-C of carbohydrates
1009	CH_2	Stretching vibrations of C-C, bending vibrations of a ring, rocking vibrations of CH_2

Table S6 FTIR values of *Chlorella vulgaris* extract.

Chlorella vulgaris [2,3]		
Wavenumber	Functional group	Characteristics
3295, 3232	O-H and N-H	OH stretching vibrations of polysaccharides, proteins and water<br>NH stretching vibrations of proteins
2966	CH_3	Asymmetric stretching vibrations of C-H in CH_3 group
2926	CH_2	Asymmetric stretching vibrations of C-H in CH_2 group of lipids and carbohydrates
2883, 2835	CH_3	Symmetric stretching vibrations of C-H in CH_3 group
1929	C=O or C=C	Bending vibrations
1452, 1383	CH_3 and CH_2	C-H bending vibrations of lipids
1326, 1270	C-N	Amide III band
1088	C-O and PO^{2-}	Stretching vibrations in carbohydrates and stretching vibrations of phosphate group
1028	C-O	Stretching vibrations of C-O-C of carbohydrates

Table S7 FTIR values of *Haematococcus pluvialis* extract.

Haematococcus pluvialis [2,3]		
Wavenumber	Functional group	Characteristics
3273, 3234	O-H and N-H	OH stretching vibrations of polysaccharides, proteins and water<br>NH stretching vibrations of proteins
2972	CH_3	Asymmetric stretching vibrations of C-H in CH_3 group
2889	CH_3	Symmetric stretching vibrations of C-H in CH_3 group
1927	C=O or C=C	Bending vibrations
1645	C=O	Amide I band
1448, 1414, 1383	CH_3 and CH_2	C-H bending vibrations of lipids
1327, 1275	C-N	Amide III band
1088	C-O and PO^{2-}	Stretching vibrations in carbohydrates and stretching vibrations of phosphate group
1039	C-O	Stretching vibrations of C-O-C of carbohydrates

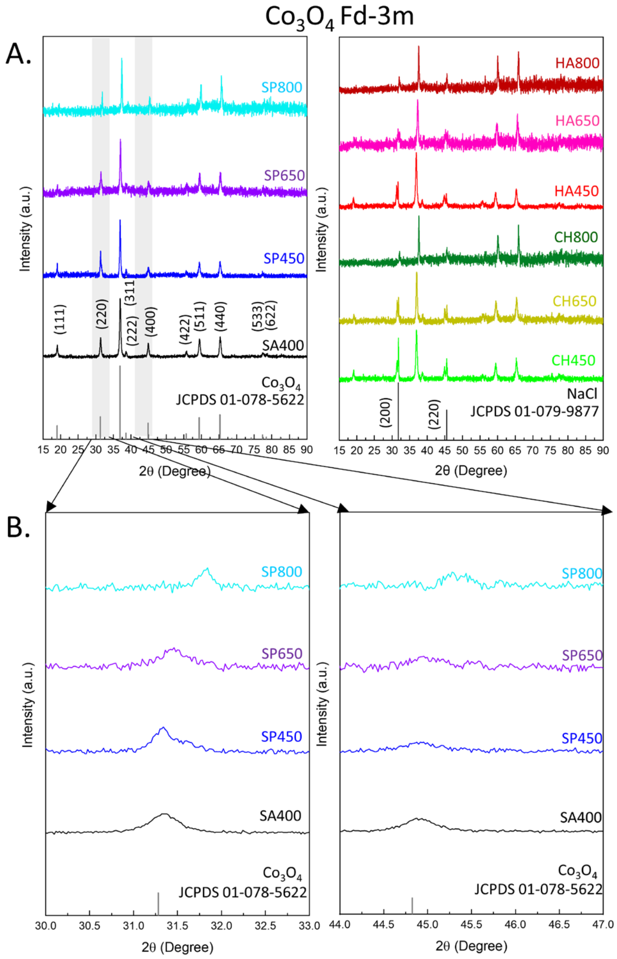

Figure S3 XRD analysis of Co$_3$O$_4$ NMs: (A) wide angle, (B) selected magnified angle regions.

Table S8 D-spacing values of SA400 Co$_3$O$_4$ NMs.

	SA400	
Miller index (hkl)	Peak pos. [°2Th]	d- Spacing [Å]
(111)	19.067	4.651
(220)	31.400	2.847
(311)	36.915	2.433
(400)	44.876	2.018
(511)	59.435	1.554
(440)	65.310	1.428

Table S9 D-spacing values for SP450, SP650, and SP800 Co_3O_4 NMs.

	SP450		SP650		SP800	
Miller index (hkl)	Peak pos. [°2Th]	d- Spacing [Å]	Peak pos. [°2Th]	d- Spacing [Å]	Peak pos. [°2Th]	d- Spacing [Å]
(111)	19.088	4.646	19.108	4.641	19.449	4.560
(220)	31.340	2.852	31.461	2.841	31.842	2.808
(311)	36.915	2.433	36.975	2.429	37.396	2.403
(400)	44.916	2.016	44.957	2.015	45.277	2.001
(511)	59.415	1.554	59.495	1.552	59.816	1.545
(440)	65.251	1.429	65.331	1.427	65.652	1.421

Table S10 D-spacing values for CH450, CH650, and CH800 Co_3O_4 NMs.

	CH450		CH650		CH800	
Miller index (hkl)	Peak pos. [°2Th]	d- Spacing [Å]	Peak pos. [°2Th]	d- Spacing [Å]	Peak pos. [°2Th]	d- Spacing [Å]
(111)	19.128	4.636	19.148	4.631	-	-
(220)	31.420	2.845	31.421	2.845	-	-
(311)	36.975	2.429	36.975	2.429	37.637	2.388
(400)	44.936	2.016	44.937	2.016	-	-
(511)	59.455	1.553	59.515	1.552	60.057	1.539
(440)	65.290	1.428	65.351	1.427	65.932	1.416

Table S11 D-spacing values for HA450, HA650, and HA800 Co_3O_4 NMs.

	HA450		HA650		HA800	
Miller index (hkl)	Peak pos. [°2Th]	d- Spacing [Å]	Peak pos. [°2Th]	d- Spacing [Å]	Peak pos. [°2Th]	d- Spacing [Å]
(111)	18.927	4.685	-	-	-	-
(220)	31.340	2.852	31.681	2.822	-	-
(311)	36.895	2.434	37.256	2.412	37.557	2.393
(400)	44.956	2.015	45.197	2.005	-	-
(511)	59.395	1.555	59.776	1.546	60.017	1.540
(440)	65.290	1.428	65.632	1.421	65.973	1.415

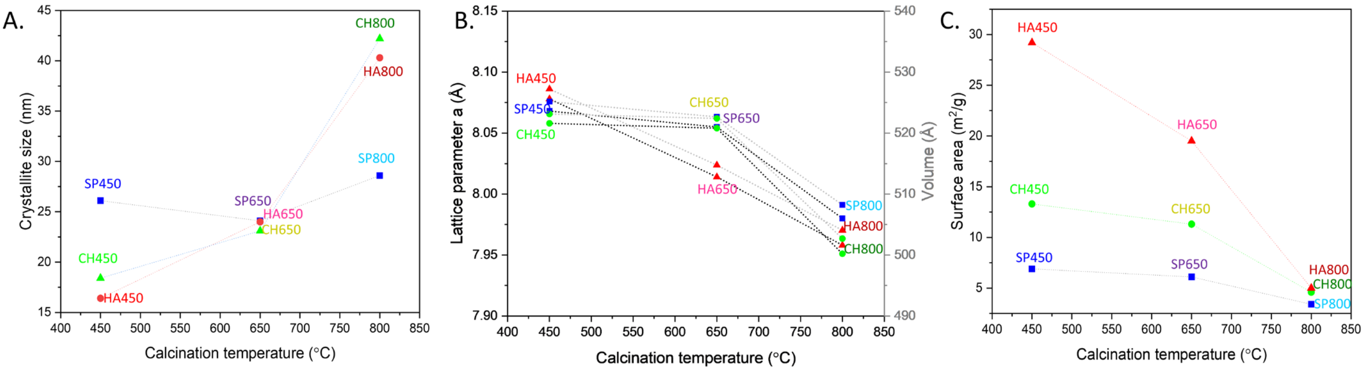

Figure S4 Influence of calcination temperature: (A) crystallite size, (B) lattice parameter and volume, (C) surface area.

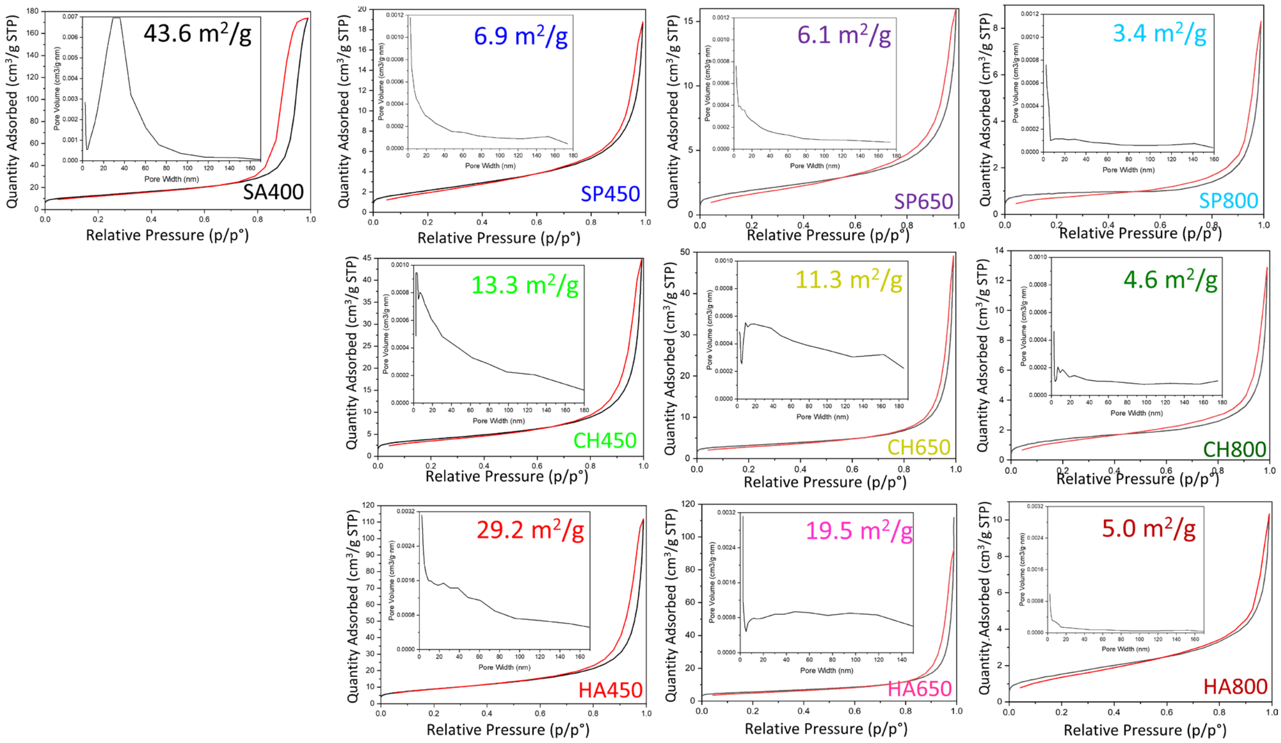

Figure S5 BET isotherms for commercial and synthesized Co_3O_4 NMs.

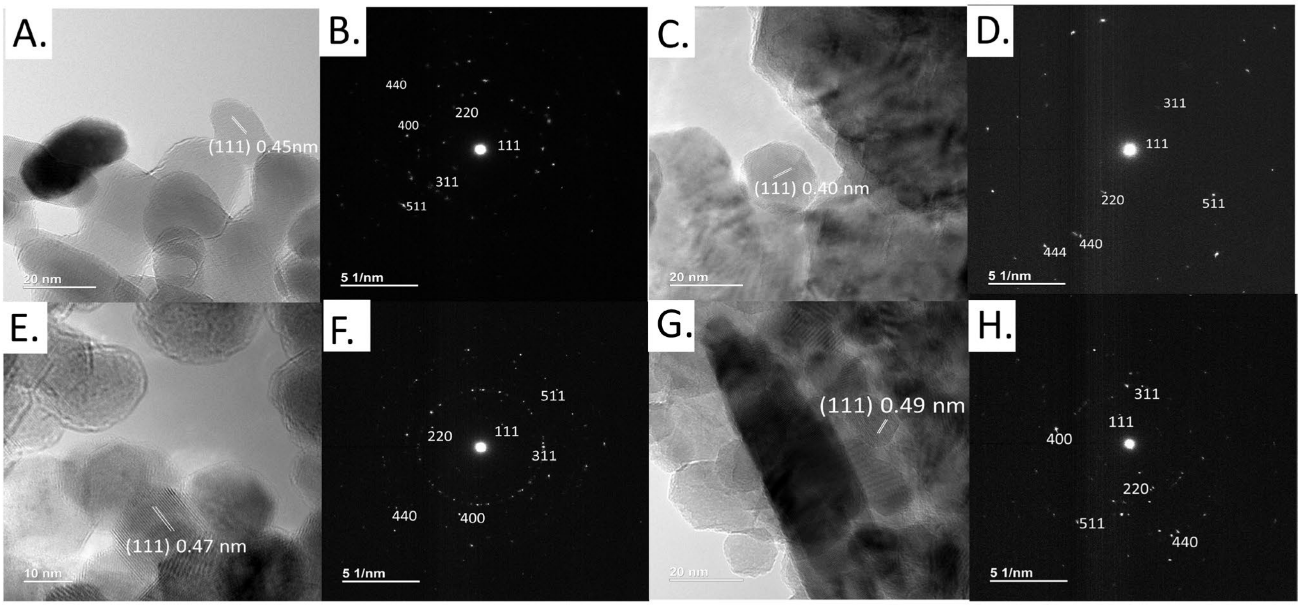

Figure S6 TEM and SAED studies of Co_3O_4 NMs: (A-B) SA400, (C-D) SP450, (E-F) CH450, (G-H) HA450.

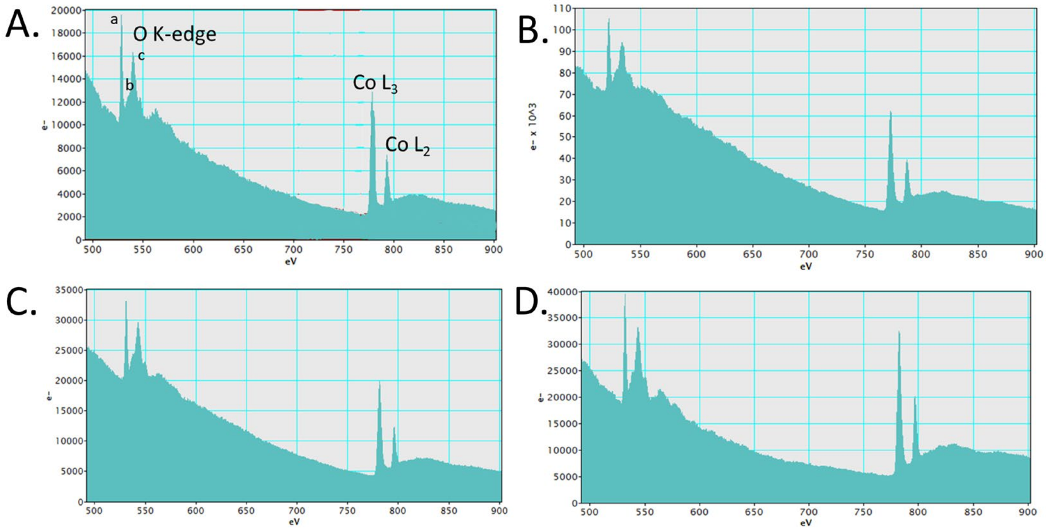

Figure S7 Raw EELS spectra of the commercial and synthesized Co_3O_4 NMs: (A) SA, (B) SP450, (C) CH450, (D) HA450.

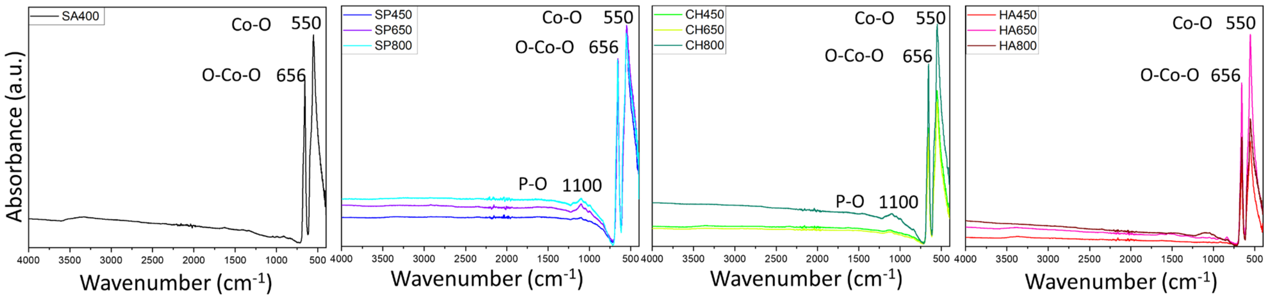

Figure S8 UATR-FTIR spectra of Co_3O_4 NMs.

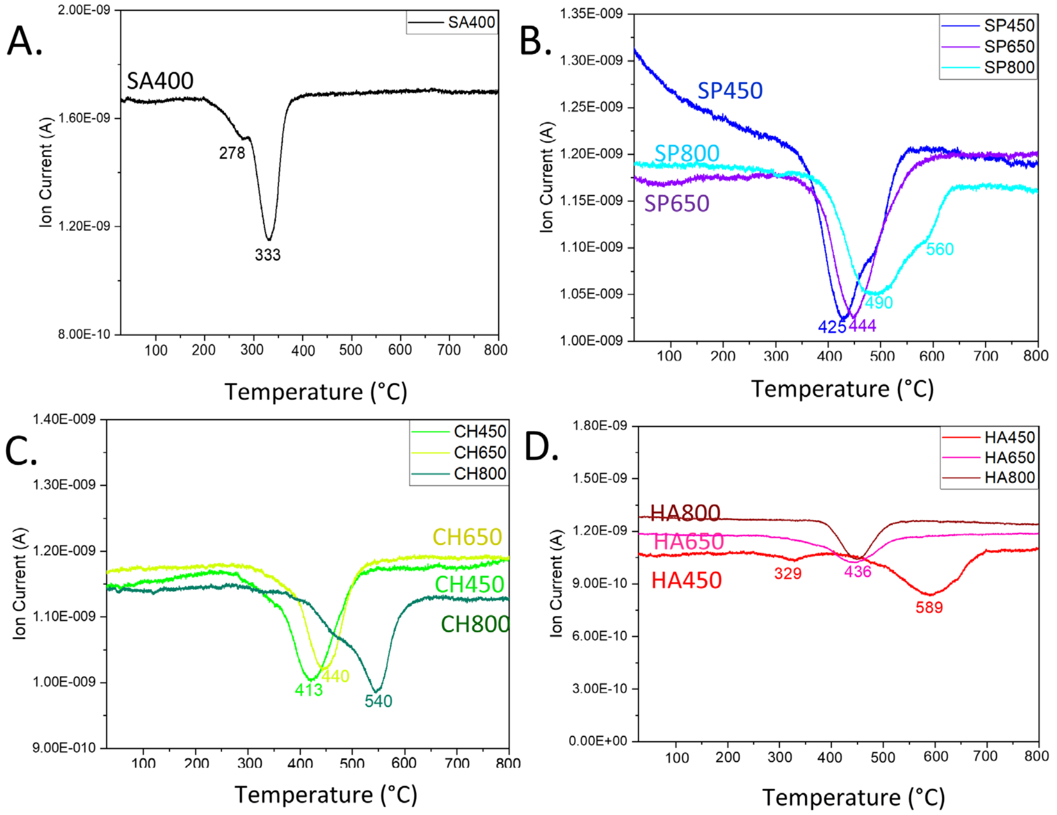

Figure S9 H_2-TPR of Co_3O_4 NMs: (A) SP400, (B) SP450, SP650, SP800, (C) CH450, CH650, CH800, (D) HA450, HA6550, HA800.

Table S12 Detected peaks in H_2-TPR and O_2-TPD (°C) for Co_3O_4 NMs.

Sample	H_2-TPR	O_2-TPD
SA400	278, 333	97, 205, 253, 314
SP450	425	130, 190, 272, 381, 428
SP650	444	111, 397, 481
SP800	490, 560	111, 471
CH450	413	107, 203, 362, 423
CH650	440	89, 211, 293, 440
CH800	540	123, 474
HA450	329, 589	105, 267
HA650	436	130, 282
HA800	436	138, 325

Table S13 DSC activation energy values (J/g) for SA, SP450, CH450, and HA450 Co_3O_4 NMs.

Sample	5% CO	5% CO + 5% O_2	5% O_2	5% CO + 5% O_2	5% CO
SA400	-14.4	-12.3	-3.6	-10.6	-6.3
SP450	-0.4	-0.3	-0.2	-0.2	-0.2
CH450	-2.4	-1.3	-0.4	-0.3	-0.3
HA450	-6.5	-2.6	-1.0	-1.0	-0.7

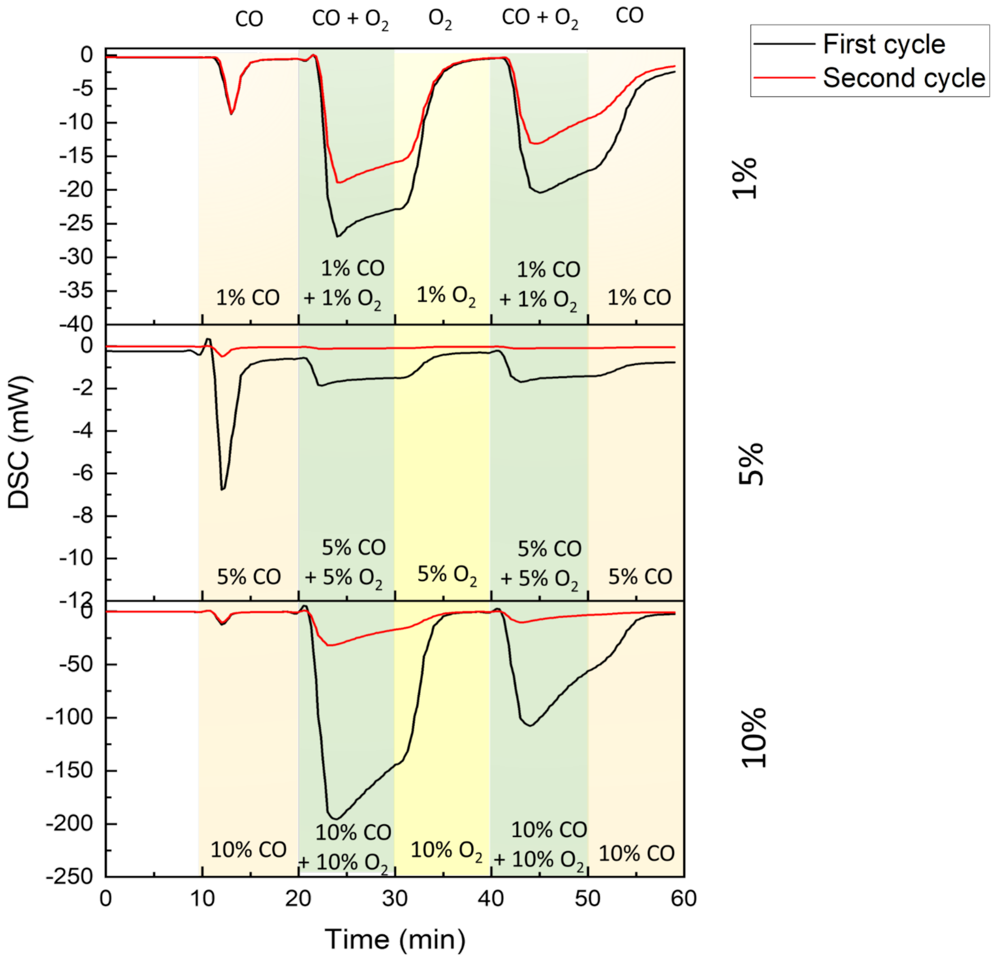

Figure S10 DSC measurements for SA400 Co_3O_4 NMs.

Table S14 DCS activation energy values (J/g) for SA400 Co_3O_4 NMs.

Gas concentration (%)	Cycle	CO	CO + O_2	O_2	CO + O_2	CO
1	1st	-15.8	-178.0	-64.2	-133.0	-68.6
	2nd	-14.8	-120.8	-47.5	-80.2	-37.7
5	1st	-14.4	-12.3	-3.6	-10.6	-6.3
	2nd	-0.9	-0.8	-0.3	-0.7	-0.3
10	1st	-17.3	-1356.8	-338.7	-670.7	-194.0
	2nd	-14.8	-204.9	-52.8	-53.2	-12.1

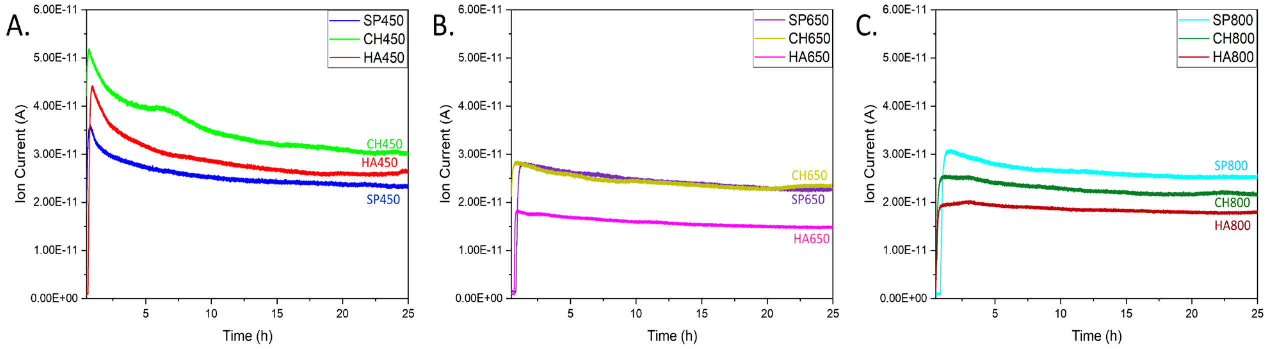

Figure S11 CO oxidation activity comparison between Co_3O_4 NMs calcined at different temperatures: (A) SP450, CH450, HA450, (B) SP650, CH650, HA650, (C) SP800, CH800, HA800.

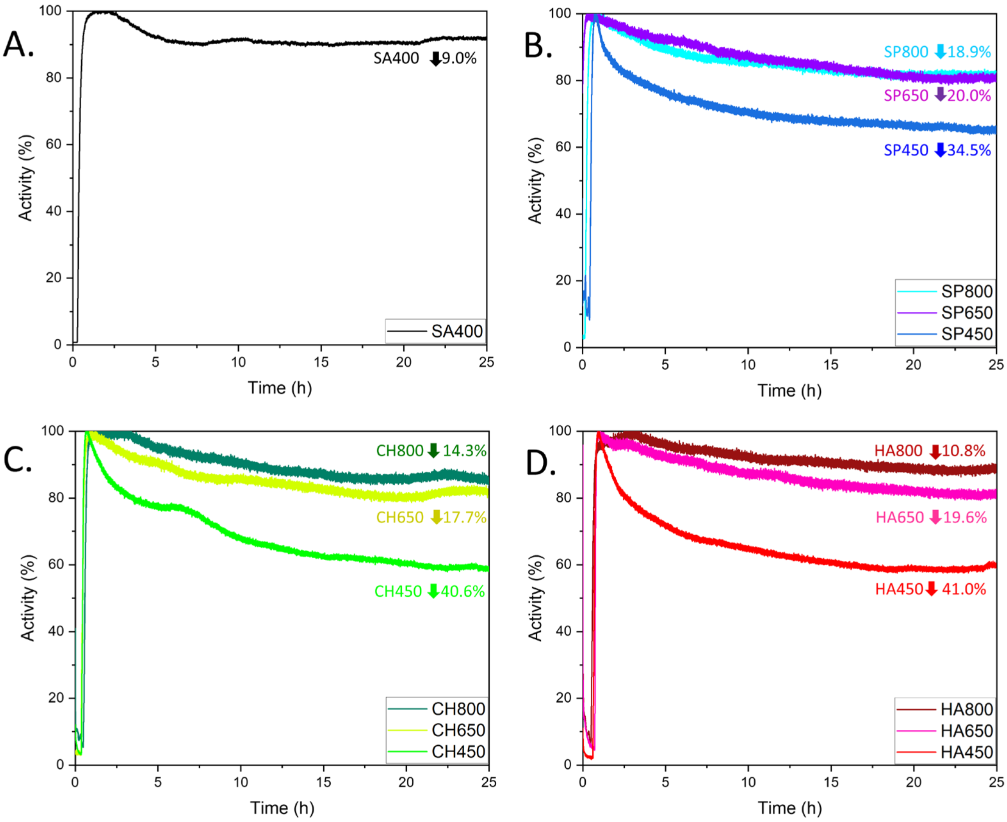

Figure S12 CO oxidation activity decrease with time of Co_3O_4 NMs: (A), SA400, (B) SP, (C) CH, (D) HA.

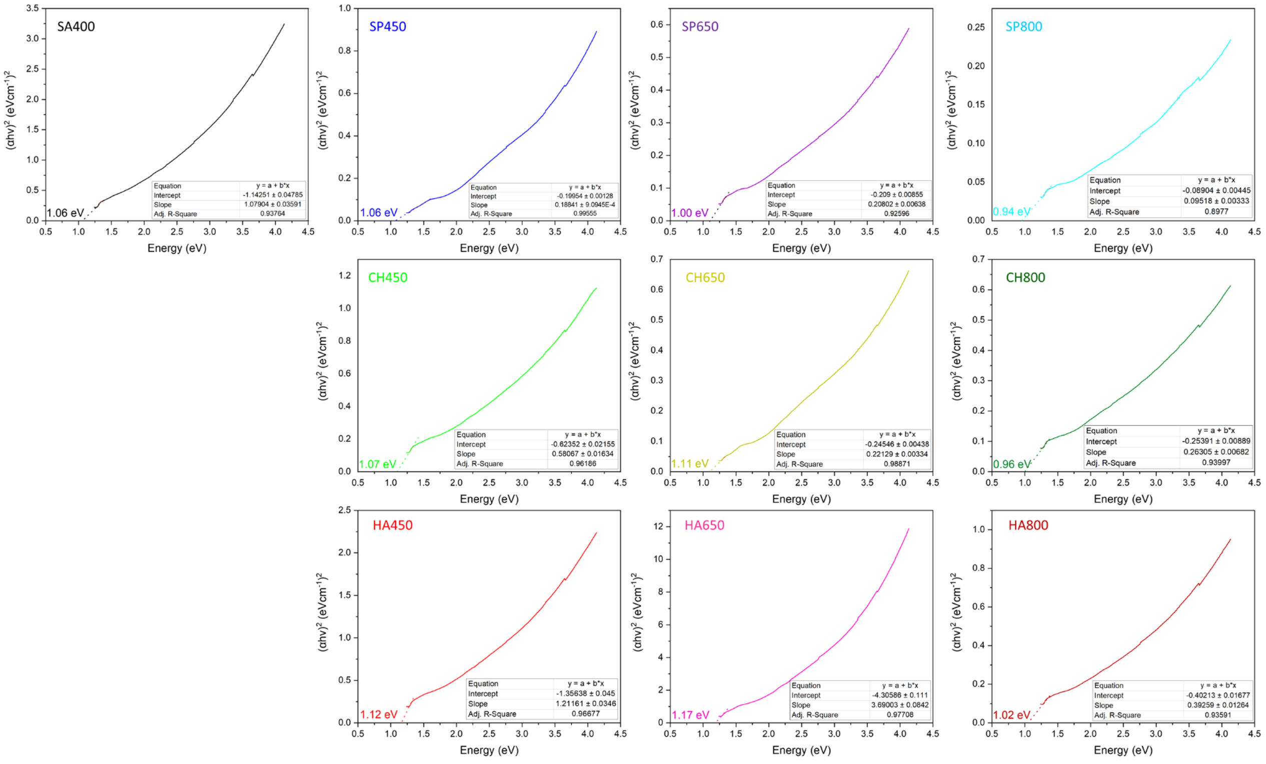

Figure S13 Tauc plots for commercial and synthesized Co_3O_4 NMs.

Table S15 Bandgap values of Co_3O_4 NMs.

	SA400	SP450	SP650	SP800	CH450	CH650	CH800	HA450	HA650	HA800
Bandgap (eV)	1.06	1.06	1.00	0.94	1.07	1.11	0.96	1.12	1.17	1.02

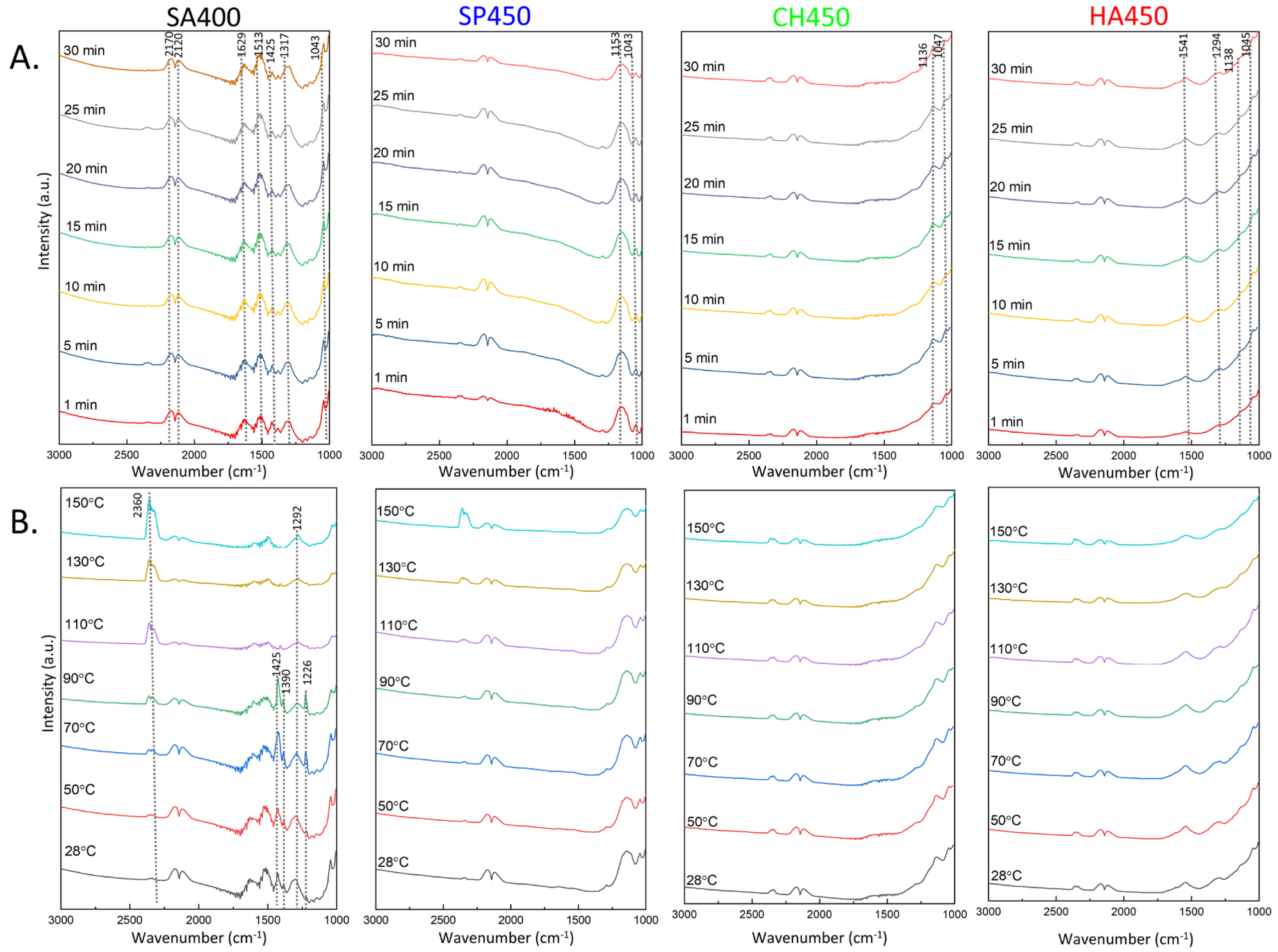

Figure S14 DRIFTS spectra of SA400, SP450, CH450, and HA450: (A) CO oxidation activity over time (carbonate formation), (B) CO oxidation activity upon heating (carbonate stability).

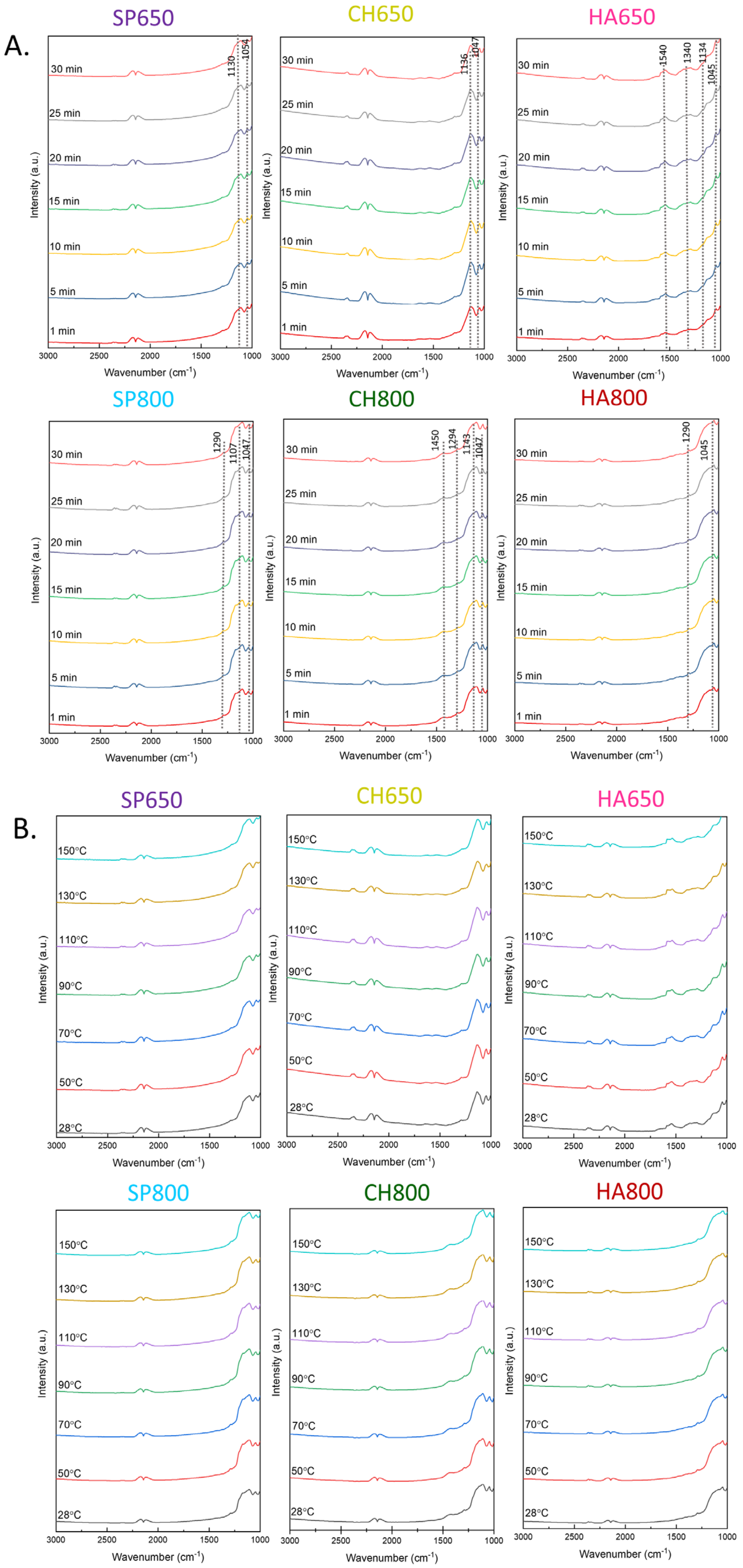

Figure S15 DRIFTS spectra of SP650, SP800, CH650, CH800, HA450, and HA800: (A) CO oxidation activity over time (carbonate formation), (B) CO oxidation activity upon heating (carbonate stability).

References

1 I. Michalak, M. Mironiuk, K. Godlewska, J. Trynda and K. Marycz, *Process Biochem.*, 2020, **88**, 129–137.

2 M. Mecozzi, M. Pietroletti, M. Scarpiniti, R. Acquistucci and M. E. Conti, *Environ. Monit. Assess.*, 2012, **184**, 6025–6036.

3 A. Poonprasartporn and K. L. A. Chan, *Biochim. Biophys. Acta - Mol. Cell Res.*, 2021, **1868**, 119024.
